# The Age of Libel—Cooper *v*. Wakley; with a Critical Examination of the Evidence, Arguments, Verdict, and Moral Consequences of This Trial

**Published:** 1829-01-01

**Authors:** 


### LXIX.

### The Age of Libel.

"Discit enim citius meminitque libentius illud,
"Quod quis deridet, quam quod probat." *Horat.*

It is not a little remarkable, that the last four years have given origin to more *libels* in the medical press, and more law-suits in consequence thereof, than the whole period of 60 years that preceded—namely, since the first introduction of medical *periodical* literature into this country! It is not our intention to trace the remote, the predisposing, or the proximate causes of this phenomenon. The fact is certain, that a kind of fermentation took place about the period in question, in the body politic of medicine, producing such intestine commotion, such warring of elements, such evolution of mephitic gases, as threatened a dissolution of all those bonds that held medical society together! We do not quite agree with those who attribute all this explosion of the worst passions of our nature to a single cause, or to a single individual. Deep and unseen operations are long going on in the bowels of the earth, before the dread convulsion rends the solid rock, opens the yawning chasm, and swallows the crashing city, with all its terror-stricken inhabitants. The fiery elements are long preparing for the conflict, before Etna's crater vomits forth the cloud of flame, or flood of boiling lava. In the treacherous calm, the quiescent atmosphere of the Antilles, when all Nature seems drooping into languid and safe repose—when not a vibration is perceptible in the leaves of the forest, nor a ripple on the bosom of the deep, the dread machinery of the Tornado is in full play, unseen by human eye, unheard by human ear, until the very moment when the aerial torrent rushes from its hidden source, and sweeps into ruin every production of nature or art on the surface of earth and ocean. It is the same with the moral as with the physical world. The seeds of discord and disaffection must long be sown in the minds of men, before the political incendiary can light the torch of civil war.

But to come nearer our subject. The distracted state of the profession—the unnatural division of its members into so many motley castes—its tri-corporate governance, if that be government, where laws exist without power or will to enforce them, and powers are exercised without reason to sanction them—the abuses which crept into public institutions, if institutions can be called public, which are not seldom worked by a secret junto, and sometimes converted to private emolument—institutions where the price of admission is not *always* the possession of talent, and where, according to the genius of our country, merit has a double meaning, viz. patronage, or wealth—but above all, the divisions and jealousies of medical society itself, split into three parties, where it should be "one and indivisible," (as far as its education and government are concerned)—without a head to direct its energies—without a heart to cherish its sympathies—without a soul to unite its interests!—these, and many other causes conspired to produce an æra that was singularly favourable to the evolution of all the angry passions of our nature, by the exposure of serious, the exaggeration of trivial, and the fabrication of imaginary grievances. But such exposures, exaggerations, and fabrications would have been deprived of more than half their zest, had they not been engrafted on *personal* satire, and wafted on the pinions of ridicule:—

"There is a lust in man no power can tame,
"Of loudly publishing his neighbour's shame;
"On eagle's wings immortal scandals fly,
"Whilst virtuous actions are but born, and die."

As hardly one in one hundred of the medical, or of any other profession, ever becomes conspicuous, whether by virtues or vices—talents or demerits; so, it requires but little insight into human nature to know, that, whoever can stigmatize, with any tolerable talent, these prominent characters, will offer a delectable treat to the "envy, hatred, and malice," of ninety-nine in the hundred. This is a law of human nature, which, no doubt, has some ultimate wise end or object—though, it is not very easy to find it out! He, however, who possesses a certain degree of callosity of nerve, and recklessness of future consequences, may turn this failing of mankind to some account! Those who consider themselves secure, enjoy the storm by which their neighbours are ship-wrecked!

"Suave mari magno, turbantibus Æquora ventis,

"E terra magnum alterius spectare laborem."

That the Lancet (we speak merely of the Journal, without reference to the secret or ostensible writers in it) did avail itself, without scruple, of the public appetite for scandal, and supply the demand to an amount hitherto unexampled in medical literature, no one will be hardy enough to deny. Personal satire (would that we were unauthorised to term it defamation) became the order of the day; and the age of LIBEL commenced—an IRON AGE, that will form no gratifying epoch in the history of British medicine! That resistance and re-action should be called forth, is not to be wondered at; and that many who were disinclined to conflicts in the war of words, should have been drawn into the vortex, from feelings of resentment, or for the purpose of self-defence, might be easily proved. We do not urge it as any extenuation of *our own sins of commission*, that men of the most peaceable dispositions, may be induced, by the irritation of the moment, to use libellous language, which, without such irritation, they would never think of employing. But this is a very different thing from the new *system* of literary warfare, in which the provocation and the libel are fired from the same cannon—and that indiscriminately, at every prominent object, save and except a small coterie of favoured individuals, who are always exempted from the failings of humanity! If retaliation is attempted, the LAW is instantly flown to by the LIBELLER—but if the law be invoked to shield the *libelled* individual, the injury is aggravated by the plea of *justification*; or, should that be impracticable, by the denouncement of unceasing persecution for daring to resist! Such a system of literary despotism, cruelty, and proscription, was never before witnessed in any age, or in any country! The GOOD which it effects, is no counter-balance to the heartless profligacy—the dereliction of all generous principle—the estrangement of every social feeling—the annihilation of all friendly confidence, which inevitably result among the members of the profession itself. —To which ought to be added, the degradation and contempt into which medical society must fall, in the eyes of the world at large, by the perpetual exposure of these calumnious combats among its members.—Should such a system continue for a few years longer (which we hope will not be the case) it is impossible not to foresee, that the profession will be deprived of all respectability, and deserted by all respectable people.

We have been led into the foregoing reflections, by a transaction which has occurred and been judicially disposed of, within the year just closed; but which will be referred to by succeeding generations, with mingled sensations of horror, and we hope of pride—horror, at the brutality of the times in which *we live*—pride, that such an iron-age has passed away!

"Quid nos dura refugimus

"Ætas? Quid intactum nefasti

"Reliquimus? *Hor.*

A young gentleman has the misfortune to be the Nephew of one of the greatest surgeons of Europe—to be educated in the best possible manner—to spend many years in the Hospital, so long the theatre of his Uncle's fame—to become a teacher of anatomy, an expert operator, and, in due time, and not before the mature age of 30 years, to be appointed one of the surgeons of the said hospital. In short, there is but one thing against him—he has given offence to the editor, or sub-editor of a medical journal, and consequently, to all the editor's hirelings. One of these hirelings has long attended the operations of the surgeon, and long witnessed his adroitness and surgical knowledge. But these are either passed over, or faintly noticed. At length, one of those untoward occurrences which, in the dark and difficult operation of lithotomy, have happened in all ages, and baffled the dexterity of the boldest surgeons that ever took gorget in hand—an occurrence that kept John Hunter an hour and a quarter in greater agonies than was his patient on the table —one of these untoward occurrences, we say, which no penetration could foresee, no dexterity avert—befel the junior surgeon, and protracted a case of lithotomy to 50 minutes duration. Every spectator who had one drop of the milk of human kindness in his composition—whose heart had not, by some strange mistake of nature, been cast in the mould of the hyena, instead of that of man—whose soul the beams of heavenly mercy, or even human philanthropy had ever warmed, would have sympathised with the operator, whose feelings might have been over-powered, whose nerves might have become unstrung, whose hand might have become tremulous—or, whose judgment might have become obscured.

But what was the conduct of some half-dozen of marble-hearted, or rather heartless spectators, on this occasion? To deny the difficulties of this operation—to distort the efforts made to overcome them—to exaggerate the sufferings of the patient—to malign the expressions, and oppugn the procedure of the operator!

We drop the pen, ashamed (for the first time) of that profession to which we have been attached—ardently attached, for nearly half a century! The following trial will contrast the refulgent and dark portions of the medical character, in this country;—and will tell to foreign nations, that we are, indeed, that strange *medley*, of which we used to upbraid our neighbours, the FRENCH:—a mixture of the tiger and monkey—with, thank God, some remains of the generous and noble LION!

### COOPER *v.* WAKLEY.

This cause, which was specially appointed for this morning (Dec. 12), excited the most intense interest. Long before the sitting of the Court, at half-past nine o'clock, the different avenues leading into the court were so crowded, that there was scarcely any possibility of forcing a passage. It was with the utmost difficulty, with the most active assistance of constables and the officers of the court, that counsel, jury, and witnesses could obtain an entrance. Almost every hospital surgeon and eminent practitioner in London was present, besides an immense number of students.

At half-past nine o'clock the defendant appeared in person on the floor of the court. Mr. Brougham and Mr. Kelly, his counsel, were also present. Sir James Scarlett, Mr. F. Pollock, Mr. Scarlett, and Mr. Platt, were counsel for the plaintiff. Only six special jurymen answered to their names. After some hesitation Sir James Scarlett prayed a *tales*. The talesmen were then called into the box, but before they were all sworn, three of the special jurymen, who had been previously called and did not answer, made their appearance and were sworn. Three talesmen were then added to the jury, and, after several of the special jurymen had been fined for non-attendance, the jury were sworn.

On the bench we noticed Sir Astley Cooper (the uncle of the plaintiff), Mr. Brodie, and Mr. Green. Dr. Roget sat to the left of Sir James Scarlett, within the bar.

The defendant, on coming into Court, brought with him a cast of a man in the position in which a patient is tied, when undergoing the operation of lithotomy. He had also a pelvis and case of instruments, such as were used on the occasion. He applied to Lord Tenterden to be accommodated with a table; but his Lordship stated, that in consequence of the pressure at the doors, it was impossible for him to have his wish complied with at present. A table was afterwards brought in.

Mr. SCARLETT opened the pleadings. The declaration charged the defendant with having published a certain false, scandalous, and malicious libel, imputing to the plaintiff, Mr. Bransby Cooper, the unskilful performance of an operation of lithotomy, which took place at Guy's Hospital in March last. The defendant had pleaded several special pleas of justification, setting forth the matter charged as libellous, and averring that the whole of it was true.

The alleged libel was contained in Nos. 239 and 240 of a weekly publication called the *Lancet*, and was in the following words: —

"GUY's HOSPITAL.

"THE OPERATION OF LITHOTOMY, BY MR. BRANSBY COOPER, WHICH LASTED NEARLY ONE HOUR!*

"We should be guilty of injustice towards the singularly-gifted operator, as well as to our numerous readers, if we were to omit a 'full, true, and particular account' of this case. It will, doubtless, be useful to the country 'draff' to learn how things are managed by one of the privileged order—a hospital surgeon—nephew and surgeon, and surgeon because he is 'nephew.'

"The performance of this tragedy was nearly as follows:—

"Act 1. The patient,† a labouring man from the county of Sussex, thick set, ruddy and healthy in appearance, and 53 years of age, was placed on the operating table, at a few minutes past one o'clock, on Tuesday the 13th. The only one of the surgical staff present, besides the operator, was Mr. Callaway. The ceremony of binding the patient we need not detail; the straight staff was introduced, and was held by Mr. Callaway. The first incision, through the integuments, appeared to be freely and fairly made; and, after a *little* dissection, the point of the knife was fixed (apparently) in the groove of the staff, which was now taken hold of, and the knife carried onwards—*somewhere*. A small quantity of fluid followed the withdrawal of the knife; the forceps were now handed over, and for some time attempted to be introduced, but without effect. 'I must enlarge the opening,' said the operator, 'give me my uncle's knife;' this instrument was given, and a cut was made with it, without the staff being re-introduced. The forceps were again used, but as unsuccessfully as before; they were pushed onwards to a considerable distance, and with no small degree of force. 'Its a very deep perineum,' exclaimed the operator. 'I can't reach the bladder with my finger.'

"Act 2. The staff re-introduced, and a cutting gorget passed along it—various forceps employed: a blunt gorget—a scoop—sounds and staves introduced at the opening in the perineum. 'I really can't conceive the difficulty—Hush! Hush! Don't you hear the stone!'—'Dodd, (turning to the Demonstrator,) have you a long finger? Give me another instrument—Now I have it! Good God! I can hear the stone when I pass the sound from the opening, but the forceps won't touch it—O dear! O dear!

"Such were the hurried exclamations of the operator. Every now and then there was a cry of Hush! which was succeeded by the stillness of death, broken only by the horrible squash, squash, of the forceps in the perineum. 'Oh! let it go—pray let it keep in,' was the constant cry of the poor man.

"This act lasted upwards of half an hour; the former upwards of 20 minutes. The stone was eventually laid hold of, and never shall we forget the triumphant manner in which the Assistant Surgeon raised his arm and flourished the forceps over his head, with the stone in their grasp. The ope-

* "The following passage occurs in John Bell's great work on surgery;—'Long and murderous operations, where the surgeon labours for an hour in extracting the stone, to the inevitable destruction of the patient.'"

† "The poor fellow, who has left a wife and six children, said, that he 'came to town to be operated upon by the 'nevey' of the great Sir Arstley.'

rator turned to the students and said, 'I really can't conceive the cause of the difficulty.' The patient being upon the table, bound, while the operator was 'explaining.'

"The man was put to bed much exhausted, but rallied a few hours afterwards, and leeches were applied, in consequence of tenderness of the abdomen. He passed a restless night, was in great pain, and was bled from the arm on the following morning. Leeches were applied in the afternoon, and about seven o'clock in the evening, death ended the poor fellow's sufferings, about 29 hours after the operation.

"EXAMINATION OF THE BODY.

"There was a very large and sloughy wound observable in the perineum, and the scrotum was exceedingly dark coloured, from ecchymosis. The finger could be passed to the prostate without difficulty, which was not deeply situate; indeed, it was the declared opinion of Dr. Hodgkin and Mr. Key, that the man had not a 'deep perineum.' The cellular tissue throughout the pelvis was easily lacerable, and this was especially the case with the portion between the bladder and the rectum, admitting of the passage of the finger with great facility, and to a considerable distance. There was a tolerably fair lateral section of the prostate and neck of the bladder. The gland itself was larger than natural, and the portion which is designated the third lobe, presented a singular appearance, being of the size of the tip of the little finger, and forming a kind of valve at the neck of the bladder; part of this third lobe had a dark-coloured appearance, and it seemed as if some substance had been resting upon it. The bladder itself presented nothing remarkable.

"The peritoneum lining the abdominal parietes was highly vascular, and there was a slight quantity of turbid serum in the cavity of the abdomen. The kidneys had a mottled appearance throughout their cortical substance.

"There are two or three points in this case to which we beg particular attention; first, the statement of Mr. B. Cooper, at the time of the operation, that he 'could not reach the bladder with his finger,' as contrasted with the fact of the bladder being very readily reached in the post-mortem examination; the man not having a deep perineum. Secondly, the circumstance of the finger passing with facility, between the bladder and rectum to a great depth, as considered in connexion with another declaration of Mr. Cooper, that he could not feel the stone with the forceps, until the time of its extraction, although a sound, passed into the bladder downwards, from the penis, struck upon the stone; as was the case also, on one or two occasions, when a staff was passed at the perineal opening.

"The surface of the calculus was rather larger than the disc of a shilling, flat, oval-shaped, and apparently consisting of lithic acid."

A long discussion arose, as to which party was to open the case; Sir James Scarlett contending that as some of the affirmative issues were thrown on the plaintiff, who was to prove his own skill, he had a right to begin; and the defendant, on the other hand, insisting that as he had charged the plaintiff with unskilfulness, and was to prove the truth of his pleas, he ought to proceed with his case before that of the plantiff's was gone into. In support of his argument, which was of some length, he cited various authorities.

Lord TENTERDEN observed, that as the decision in this case might be quoted hereafter as a precedent, he would take the opinion of his learned brothers who were sitting in the Bail Court upon the question. His Lordship then retired, and on his return in about five minutes, stated his opinion to be (in which the other judges concurred) that the defendant had a right to begin. The plaintiff, as a surgeon, was to be supposed to be a skilful person until the contrary were shown, and therefore there was no necessity for him in the first instance to go into evidence to establish that fact; and as the defendant had pleaded the truth of the matter which imputed unskilfulness to the plaintiff, it was incumbent on him, in the outset, to prove the truth of his allegations.

Sir J. SCARLETT trusted, as that was the decision of the Court, that whatever the termination of this case might be, he should be at liberty to examine the plaintiff's witnesses. It might happen that the defendant's case would fall to pieces, and then he (Sir J. Scarlett) should insist that his evidence ought to be heard for the purpose of proving that the operation had been performed with the utmost skill. He should ask, on behalf of the plaintiff, a gentleman of high honour, and who was greatly esteemed, both in and out of his profession, that he might have an opportunity of showing that the calumny which had been attempted to be fastened upon him was without foundation. He mentioned this now, in order that, in the event of the defendant failing to prove the whole of his pleas of justification, it might not be said that the plaintiff had no right to go into evidence in vindication of his professional character.

Lord TENTERDEN did not think it necessary for him to give an opinion upon that point at present.

The defendant then suggested that the witnesses on both sides should withdraw.

Sir J. SCARLETT had no objection to the withdrawal of those witnesses who spoke to facts, but those who were to give opinions he considered it essential and necessary that they should remain in court.

The defendant said, as he could not at that moment make a separation of the witnesses who were to speak to facts, and those who were to give opinions, he would not insist on the withdrawal of any of them. They might remain, if the Court pleased.

The defendant then addressed the jury. They had already heard from the learned gentleman who had opened the pleadings, that this was an action instituted against him (the defendant), the editor and proprietor of the *Lancet*, for an alleged libel of and upon the professional character of the plaintiff. It was stated in the declaration that he had published a report of a "supposed" operation at Guy's Hospital, falsely and maliciously, and it was inferred from the declaration that no such operation was performed in that institution; and that what he had published was nothing more nor less than gross calumny. Guy's Hospital, as the jury must be aware, was an institution of very great importance, not only as an institution of charity, but one from which it was expected that there should emanate the first principles of his (the defendant's) profession, practised in the very first and best manner. It had attached to it an extensive medical school. It had lectures there, and a very large attendance of students. The practice which the students witnessed in the institution was necessarily carried by them to the most distant parts of the kingdom. Hence it was of the utmost importance to the public welfare that the practice there inculcated should be calculated to promote the interests of the public, and alleviate, as far as it was possible, the sufferings and miseries of mankind. Guy's Hospital was founded solely by one individual, Thomas Guy, in the year 1722 or 1724, and he at that period left to the Institution a sum equivalent to 200,000*l.* consequently the funds of the Institution, from the increase that had taken place in point of value, were immense. Of course it became of great consequence that these funds should be appropriated in the best manner—that individuals of the greatest possible skill should be elected to fill its situations, both medical and surgical, and it was not fair that those offices should be filled in any other manner than what was consonant with the intention of the founder and would be of the greatest benefit to the public. The plaintiff, Mr. Bransby Cooper, was one of the persons who had been elected to fill the office of surgeon, and in the duties of his situation he performed the operation which was published in No. 339 of the *Lancet*. That journal was projected by him (the defendant), and was first published in 1823. He considered that by publishing the lectures delivered in public institutions, he should be able to place in contrast the theories of the different individuals in the lecture-room, and the practice in the wards of the hospital; and this he thought was of immense importance, for, by so doing, the lecturers were stimulated to a greater exercise of their duty, as their opinions were laid before the public, who had an opportunity of seeing what those opinions were, and, at the same time, of seeing whether the practice used in the wards was a practice of neglect or attention. The publication of lectures had led to a good deal of discussion in the courts of law. The publication of hospital reports had led to great benefits, as regarded the public, who had thus the advantages of the opinions of hospital surgeons; and they had to endure the scrutiny of the public, and could not hack and halve the individuals with impunity. He was of course under the necessity of employing a great number of reporters, who were in the practice of taking notes of the cases admitted, and carefully registering all the points connected with them. The reporters he had employed, as far as he had been capable of judging, were men of the most honourable character, and had fulfilled their duty in a very accurate and conscientious manner. The reports furnished by them he was of course compelled to rely on, as all editors were, for their correctness. They were transmitted to him regularly from the various institutions. The report of this operation was sent to him by a gentleman of very high character,—a gentleman whom he should call into the witness-box to-day, and who would himself state that he witnessed this operation, and that the report which he furnished was in every respect correct. He (the defendant) would assert this openly before the Court, and he challenged every inquiry—every strict and scrutinizing investigation—into the reporter's character. He had heard, indeed, that attempts would be made to cast some imputation upon the young man, but he defied calumny, and courted scrutiny. When the report was transmitted, as he found it one of an extraordinary character, and as it referred to circumstances of a still more extraordinary character, he paused before he inserted it. He did not publish it in the first number of the *Lancet* which appeared after it occurred, but waited till the period of publishing a second one arrived. When the report was first brought to him, it contained some statements against the operator rather harsher than those which it now contained. The reporter considered it his duty to characterize such an operation by the strongest terms of reproach; and he stated, on his honour, before the report was published, that it was correct in every particular. Upon that assurance, therefore, he (the defendant) considered that he had no other course to pursue in his public character than to present it to the public, whatever the consequences might be. Having made a few alterations in it with regard to the expressions he had mentioned, and introduced the phrases, "nephew and surgeon, and surgeon because he is nephew," he inserted it word for word as he had received it. He should prove these facts, and had little fear of his case "falling to pieces," as his learned opponent had stated. He believed the learned gentleman would have ample reason for producing all his witnesses, and giving such evidence as he could adduce as to the skill of the plaintiff. If it could be proved that the report was incorrect, nothing would give him greater regret than to find that he had done an injury to the plaintiff; for it was not by such means that the profession was instructed; but when men came boldly forward to sanction such proceedings as these, it gave a stain to the profession which the events of ages could not remove. When men came forward to swear that this operation was performed skilfully, he knew what they would swear, and it must be presumed that they were unable themselves to perform the operation in any other way. With these observations he should leave the case in the hands of the jury. He should call a great number of witnesses to prove that the report was, in every respect, correct; and if they (the jury) were satisfied upon that point, he should of course be entitled to their verdict.

The defendant then called and examined the following witnesses.

Mr. Holdiman Partridge.—I reside at Colchester, and am a member of the College of Surgeons. I have been in practice rather more than 14 years. I have witnessed many operations in lithotomy, and have performed them myself 16 or 18 times. I witnessed the operation performed by Mr. Bransby Cooper at Guy's Hospital in March last. I have read the report of that operation in the *Lancet*. It struck me at the time to be correct, and I have had no particular reason to alter my opinion since, though I did not examine it very minutely. The patient appeared to be a *very healthy man*. I remarked it at the time. I think Mr. Cooper himself introduced the staff; but the second incision was made without the staff. After the first external incision, all instruments were withdrawn. (The defendant here produced a figure representing the situation of the patient, which the witness deposed to as being correct.) The hands of the patient were tied to his feet, and his knees to his neck, as represented by the model now produced. The patient remained in that position nearly an hour. During that period a sound was repeatedly introduced. Several cuts were *attempted* to be made into the bladder with a knife. This instrument (*a cutting gorget*) was introduced into the wound. A blunt gorget was also introduced, and the scoop and several pair of forceps. During the operation the patient called out several times to the operator to desist. *The operator stated several times that he could not explain the difficulty.* He appeared to be perplexed and hurried in consequence of the long delay. He did not appear to act with any regular scientific design. He introduced his *finger with some force*, but it did not strike me as being very violent. *He used the instruments in the ordinary way*, and varied them according to the different purposes, but failed in lighting upon the stone. I don't consider that the forceps entered the bladder the first time. The impression on my mind was, that the opening in the bladder was not sufficiently large to get the forceps in; *but I think there was an opening, because I saw a discharge of water and blood.* The operator said that he felt the stone when he passed his staff through the urethra, and could also feel it when he passed the sound through the incision in the perineum. He also said that he could not feel it with the forceps. The reason of this was, that the forceps, if straight or slightly curved, would pass under the stone, which was high up in the bladder. Mr. Cooper made many attempts to feel the stone with his finger. *He left his seat and measured fingers with those of other gentlemen to see if any of them had a longer finger.* I cannot say that I think Mr. Cooper performed the operation in a scientific manner. I do not think that it was performed in such a manner as the public have a right to expect from a surgeon of Guy's Hospital. The average time for performing operations of this description is four or five minutes. The operation in question occupied, I think, nearly an hour. After the staff had been introduced, and the first incision made, Mr. Cooper used a straight staff with a knife. When he found he could not introduce the forceps on the first attempt, he withdrew them, and made another cut with the knife without the staff being introduced. This is not the customary mode. The scoop as I have always understood, is introduced to extract those fragments of the stone that may have crumbled off. There were no fragments in this case that I saw. Twenty-five or thirty minutes is the longest time that I have known an operation of this kind to last. The average time is about five minutes. In the cases I have mentioned lasting 25 or 30 minutes, there were evident causes why the operation should last so long. Those were where the stone was large, and where it would be dangerous to enlarge the wound for fear of injuring the rectum, and there the time was lost *in drawing at the stone gradually.* In the operation in question, the stone was a small one, being not larger than a common Windsor bean, flat and round. It might have weighed about two drachms or less, but certainly not more. Stones weighing several ounces have been successfully removed. Unless the incision was large enough to admit the forceps, that instrument could not lay hold of the stone without also catching the integuments of the bladder. *The stone lay*

*above the pubes*, for the sound always touched it on being withdrawn, and it was extracted by pressure above the pubes, and *with a curved forceps*. If the operator had been aware of the situation of the stone, he should have taken these measures at first. He should have ascertained this in the first instance.

Cross examined by Sir J. SCARLETT.—I never saw the defendant before this day, nor his attorney in the cause before last night. Mr. Callaway was the assistant surgeon on the occasion, and I believe him to be a man of skill. I have had several cases where the stone lay above the pubes, and always extracted it in the manner which was at last successfully adopted by Mr. B. Cooper. The cut is made in the perineum, and the object is to get the knife into the groove of the staff, by which time it has penetrated a portion of the urethra. Then the staff is brought forward into a parallel position with the knife, and on a line with the bladder, in order to make a larger incision; the staff is then allowed to remain, and the finger is introduced in order to ascertain the wound you have made.

Sir J. SCARLETT.—You then introduce the finger and feel for the stone, after finding which, you introduce the forceps along the finger, and lay hold of the stone?

Witness.—No: in order to do that you must make too large an incision, or else have a most extraordinary small pair of forceps. At the time of the operation *I was sitting in a chair immediately behind Mr. Cooper*. I never saw Mr. Cooper before that day. I have no doubt but that the first incision penetrated the bladder. I have read the report in the *Lancet*, but I never corresponded with that publication. I take it in and read it weekly.

Sir J. SCARLETT here read from the libel the following sentence:—"The first incision, through the integuments, appeared to be freely and fairly made; and, after a *little* dissection, the point of the knife was fixed (apparently) in the groove of the staff, which was now taken hold of, and the knife carried onwards—*somewhere*." The learned counsel asked the witness whether the word "somewhere" did not mean to convey an idea that the knife did not go into the bladder?

Witness.—I think it means to convey an idea that it might or might not have entered the bladder. *I do not know whether the operator would be best judge of whether the forceps entered the bladder or not:* it would depend upon what sort of an operator he was. (A laugh.) I am not prepared to swear that the forceps were a second time used with considerable force. I will neither swear to, nor contradict it. I mentioned my opinion of this operation to several persons, but I cannot now say to whom.

Sir J. SCARLETT then read the following sentence, and asked the witness whether the statement it contained was correct.—"The forceps were again used, but as unsuccessfully as before; they were pushed onwards to a considerable distance, and with no small degree of force."

Witness.—I am not prepared to swear to the truth of this. I cannot comprehend it.

Re-examined by the defendant.—The staff was introduced a second time. It would not have been necessary if the first incision had been large enough. I have never seen the defendant before this day, that I am aware of.

Mr. John Clapham examined.—I reside at Thornhill, near Peterborough, and practise as a surgeon with my father. I am a licentiate of the Apothecaries' Company. I have studied surgery at St. George's Hospital. I witnessed the operation of lithotomy performed by Mr. Bransby Cooper at Guy's Hospital. The report in the *Lancet* is correct as far as I recollect. The patient appeared a healthy man, and a favourable subject for the operation. On the withdrawal of the knife there was a small quantity of fluid, I can't say of what sort, but I suppose it was urine. I saw no gush of urine subsequently. Mr. Cooper used a knife to enlarge the openings before he introduced the forceps. The forceps were introduced more than once before the second cut. I had never seen that done before. The forceps were introduced with much force, and the operator did not appear in a state of self-possession. He said he could not reach the bladder with his finger. Great force was used with the hand. More than one gorget was used. He introduced sounds and staves at the wound in the perineum. I never before saw the gorget employed after the knife had been used to cut into the bladder. A scoop was employed. There were no fragments extracted in this case. The operator stated, in the presence of the patient, that he could not understand the case. I never before heard a surgeon speak of the difficulties of the case in the presence of his patient. He said he could feel the stone with the sound, but that he could not feel it with the forceps. I heard the staff strike the stone. The reason why he could feel the stone with the sound, and not with the forceps, was, that the narrowness of the opening would not admit them. I did not hear Mr. Cooper ask Mr. Callaway if he had a long finger, but he measured fingers with Mr. Dodd. I never saw an operator act in a similar manner while his patient was bound on the table. He appeared confused; his movements were hurried, *he used the various instruments out of their accustomed order*. The operations of lithotomy which I had before witnessed have occupied from two to six or eight minutes. I never saw any that lasted for a longer period than eight minutes. Mr. Cooper's operation occupied an hour. I never before witnessed an operation in which so much violence was used; and I do not believe it possible that the patient could recover after such an operation. I am aware of no circumstance in the anatomy of the parts which was calculated to render the operation so tedious. The stone was not so large as a walnut. The blood had ceased to flow from the external wound before the operation had terminated. The parts appeared bruised.

Cross-examined by Sir JAMES SCARLETT.—I was 20 years of age last January. I have not yet finished studying, and am still a pupil of St. George's Hospital. I have had no explanation of these matters since I came to London. I had no string of written questions put to me, and I have examined no models. I went from curiosity to see the operation performed by Mr. Cooper. The surgeon who performs the operation of lithotomy has always a variety of instruments near him. I have witnessed about half-a-dozen operations of this kind. I was on the third or fourth row from Mr. Cooper, a little to his left hand. A great number of persons were present; there might be as many as 200. I should think Mr. Callaway was a competent judge of operations of this kind. When the first incision was made, it did not reach the bladder. There was a small quantity of fluid, which I supposed to be urine, before the forceps were introduced. I am of opinion that the knife did not reach the bladder. I am a licentiate of the Apothecaries' Company. I am not 21. My representation of my age was not correct to the Company.

Sir J. SCARLETT.—You mean it was false?

Witness.—Yes.

Sir J. SCARLETT.—Did you not swear it?

Witness.—No, I gave my certificate.

Sir J. SCARLETT.—Are clergymen in the habit of giving false certificates?

Witness.—No.

Sir J. SCARLETT.—Where, then, did you obtain it?

Lord TENTERDEN said he must stop the examination. When he found a person obtaining his licentiate by means of a false certificate, for which he was liable to be indicted and punished, it was his Lordship's duty not to allow him to proceed further in his evidence.

The witness then withdrew.

Mr. Joachim Gilbert examined.—I am a member of the College of Surgeons. I was at Guy's Hospital in March last, and witnessed part of the operation of lithotomy performed by Mr. Cooper. I was present about 35 minutes. I could not endure witnessing any longer the manner in which the operation was performed. The operator used much violence,—I should say great and unnecessary violence. *He used the instruments in the accustomed manner of other operators.* I saw the staff introduced by Mr. Cooper: Mr. Callaway was desired to hold it on the left side of the patient, and then Mr. Cooper made what is called the external incision, the cut, which he did very properly; then, after making the second incision, he carried his knife forward, *and, I should say, held his arm too high;* and he then carried his knife forward, between the bladder and the fundament. A flow of blood followed. He then put his finger into the wound and passed in a pair of straight forceps on his finger. He attempted to extract the stone, but failed. He then passed in the forceps four times following, but did not succeed in extracting the stone. He then passed his finger again into the wound, *and in so doing used great violence.* In withdrawing the forceps, a squashing noise was heard. He then called for a crooked pair of forceps, which he passed upon his finger into the wound, and poked them about *in the wound.* In so doing he used great violence. He then withdrew them and passed them in a second time. He again withdrew them, and passed them in a fourth time, and he then called for "Sir Astley's knife," (a laugh) and made a cut with it, and passed his finger into the wound; *and in so doing used violence, twisting the finger about in the wound.* He did not succeed in extracting the stone; and he then appeared to be very much confused. His hand shook a great deal. He was very pale, and his lips were very white. At the expiration of 35 minutes, I quitted the theatre. My impression was, that the operation was very badly and very improperly performed. I have witnessed at least 20 operations of this kind. I never saw any last longer than from seven to ten minutes; and I have seen the operation performed in less than a minute.

Cross-examined by Sir JAMES SCARLETT.—I carry on my business at Beaminster, in Dorsetshire. I am an assistant to Mr. Phelps, who married the defendant's sister. I came to London at the time in question to pass the College. I am a pupil at Guy's, and I went there to be instructed. I did not see the report in the *Lancet* before it was printed. The first incision did not and ought not to reach the bladder. You are to avoid the urethra on making the first incision. The second cut, which ought to have reached the bladder, *did not reach it, but went between the rectum and the bladder.* I think it did not reach the bladder, and my reason for saying so is, that there was *no flow of fluid.* The forceps were thrust in with *great and unnecessary violence(!!!)*

Sir J. SCARLETT.—As if he meant to stab the man. Witness—*Yes.* (A laugh.) I was on the first row of benches. I went away, not being able to witness the operation out. I never made any communication to the *Lancet*, nor do I know the extent of its sale. I never performed the operation of lithotomy. I have witnessed at least 15 operations of that kind at Guy's Hospital, but not one of them were performed by Mr. Cooper.

In answer to some further questions by the defendant, the witness said, he had seen the plaintiff perform other operations, and *he did not consider him a skilful operator(!!)*

By Sir J. SCARLETT.—I should say that he is an unskilful operator, and that *it would be a great benefit to the public to drive him away from his situation at Guy's Hospital.*

Sir J. SCARLETT.—Have you ever witnessed the operation of tying the subclavian artery?

Witness.—I have heard of the operation, and have seen it performed once, by Mr. Cline.

Sir J. SCARLETT.—Which operation do you think requires the greatest skill upon the part of the surgeon, tying the subclavian artery, or lithotomy?

Witness.—I should think the operation of *lithotomy requires the greatest skill.*

Sir J. SCARLETT.—Then you think it would require no skill to tie the subclavian artery?

Witness.—It would require skill to do so, but more skill to perform the operation of lithotomy.

Sir J. SCARLETT.—Have you attended any lectures since you came to town?

Witness.—No.

Sir J. SCARLETT.—I mean, none at Wakley's. (A laugh.)

Witness.—No. Mr. Wakley called at my lodgings yesterday morning, and stopped for a few minutes; but he did not even sit down, and that is the only conversation I have had with him since I came to town.

*The Defendant.—Do you consider that a very ignorant surgeon might, by accident, tie the subclavian artery with success?*

*Witness.—I do.(!!!) (A laugh.)*

Mr. John Thomas examined.—I witnessed the operation of lithotomy performed by Mr. Bransby Cooper. I have not read the report in the *Lancet.* I am demonstrator of anatomy at Mr. Slee's school. Speaking, according to my impression, I think I never saw an operation performed so unscientifically, and in so bungling a manner, as that performed by Mr. Cooper. I have never spoken to the defendant before to-day, *and I don't even now know his name.*

Cross-examined by Sir JAMES SCARLETT.—Mr. Slee's school is in Dean-street, in the Borough. Mr. Slee is assistant-surgeon to the Western Hospital, a newly-erected institution, which Mr. Slee established himself.

Sir J. SCARLETT.—Suppose the defendant to be the author of the *Lancet*, I want to know how it was that he came to know your opinion on this operation?

Witness.—*I confess I was rather surprised at it myself.* I was not subpœnaed till a late hour last night.

Sir J. SCARLETT.—Do you know, now, how it was?

Witness.—*Yes.* In conversation with a pupil named Rainsford, I made the remark that I had seen the operation, and that I thought it was performed in a bungling and unscientific manner. He has told me to-day that he communicated this fact to Mr. *Wakley. I have made communications* to the *Lancet. I have made four—three of which were inserted. Those communications were at long intervals, and I did not put my name to them.* I arrived at the theatre after the incision was made into the bladder, and I stayed about 35 minutes, during which time the operation was going forward. I have been demonstrator at this new school since last October. I was present at three operations of lithotomy at Guy's Hospital.

Mr. Jeffry Pearl examined.—I witnessed the operation performed by Mr. Bransby Cooper. I have read the report in the *Lancet*, and I am not aware of its being incorrect, except that Mr. Cooper asked for "Sir Astley's knife," and not for "my uncle's knife," as stated in the report. There was *no gush of urine as usual, but merely a trickling.* I sat about the middle row, on the operator's right hand. (The witness was examined to various minute facts, deposed to by some of the preceding witnesses, and, in part, corroborated their testimony. He also spoke to the violence used as described by those witnesses, and stated, that *three fingers at once were introduced.*) I could, I think, sitting where I did, *distinguish between a small portion of arterial blood, and a mixture of venous blood and urine*—arterial blood comes in bursts. After the staff and knife were pushed forward, I believed that there issued a small portion of both *venous blood and urine.* There was not a gush of fluid at any subsequent period. I rather think that Mr. Cooper attempted to introduce the forceps after the first incision, and that, failing in being able to do so, he made a second incision. The same forceps was introduced repeatedly and *great force* was used. The operator *opened and shut the forceps with great violence.* (!) The forceps was a curved one, and it was pushed in to a considerable distance. The fingers of the operator were introduced and turned in the incision. I have witnessed, I believe, 20 operations in lithotomy, and none were performed in the same manner as this. The usual time for the performance of an operation is from four to five minutes. I saw one by Mr. Green *which lasted nearly an hour;* but that was a peculiar case: the patient had been operated on twice before, and the cicatrix was hard and uneven, and he had two very large stones to extract, which crumbled into innumerable small pieces in the bladder. The time was there occupied in removing the fragments. There was no force used by Mr. Green, and his manner of using the scoop and forceps was decidedly contrary to that of Mr. Cooper. The stone in the case of Mr. Cooper was about the size of a Windsor bean. I heard Mr. Cooper say, in the hearing of the patient and the pupils, that he could not explain the cause of the difficulty. He turned round to the pupils and said, "I can conceive no earthly difficulty against my extracting the stone." He said "Hush, hush; I can hear the stone, but cannot extract it when I apply the forceps." I think, continued the witness, that he might have taken the stone, had he introduced the forceps *scientifically.* The sound might have passed through a hole too small to admit the forceps. Mr. Cooper did not appear to me to be in a state of self-possession, and I think that he used his instrument without *any rational object.* The operation lasted about an hour. I saw the parts after the death of the patient, and I could not discover any cause in the formation to account for the delay in the operation. When I saw the bladder, there appeared to me to be *two incisions in it.* A portion of the neck of the bladder was *between the two incisions.* (!) The incisions were oblique, and not horizontal, as any incision ought to be in such an operation. *I think it utterly impossible* that the patient could have survived after *such an operation* as this one. The bladder appeared to be very thickened from *violent inflammation.* Bruises in the bladder would be likely to produce great disorganization and inflammation. I saw Mr. Bransby Cooper operate, and I should not conceive him to be *a good operator by any means.* I have been a pupil of Guy's Hospital more than a year, but am not a member of the College of Surgeons.

Cross-examined by Sir J. Scarlett.—I have been attending the hospital a year. I commenced in October, 1827. I was apprenticed at Woodbridge to an army surgeon. I never performed lithotomy myself. I continue at the hospital now. I have seen most of the operations at the hospital. I never saw Mr. Bransby Cooper perform an operation skilfully except one, and that was tying the subclavian artery. I consider that to be a difficult operation; *but it may occasionally be performed by an unskilful operator.* Mr. Laundin handed the instruments to Mr. Cooper; Mr. Callaway stood next. I read the report in the *Lancet* on the day it came out. Mr. Cooper did not use the phrase "My uncle's knife," but I have heard him repeatedly say "Fetch my uncle's *gargle*, or my uncle's *mixture*." (A laugh, in which Sir Astley Cooper joined.) It was Dr. Hodgkin's duty to dissect the body, as he conducts the *post mortem* examinations. A gentleman of the name of Lambert introduced me to the defendant. I saw him at Mr. Lambert's house; the conversation turned upon this subject, *but I did not know from that or any other conversation that Mr. Lambert was the person who furnished the report.* The tone of the conversation did not assume that Mr. Lambert was the reporter. I shall not say whether I suspected it or not. I certainly did not suspect it from that conversation. I suspected Mr. Lambert because he was generally suspected. I have been at his house three or four times. I never met the defendant there above once. A pupil was present when I saw the defendant. The defendant asked me whether the report was correct. *The defendant and Mr. Lambert endeavoured to show that the forceps had passed between the bladder and the rectum.* They did not endeavour to persuade me to state that fact, *but they gave very good reasons for their believing it.* Mr. Whitaker was present, but no other person. I was there an hour. No model was produced to assist my reasoning. I called at the defendant's house last Monday, and stayed only ten minutes. There was a conversation as to the time of the trial coming on. I am not competent to say whether or not the forceps passed between the bladder and the rectum. I had not formed any opinion on the subject, but I can state that the operation was not scientifically performed. I believed that at one time *the forceps were between the bladder and the rectum.* On seeing the parts after death, *I observed Mr. Lambert had his hand between the bladder and rectum, and took the part up to Dr. Hodgkin, and said, "Doctor, here's an opening." I did not*

*hear Dr. Hodgkin say, "Thou hast done it thyself." I do not believe Mr. Lambert did it.*

Re-examined.—The defendant did not persuade me to give any evidence in this cause. He asked me to read the report again, that I might be satisfied of its correctness.

Mr. James Lambert stated that he was present at the operation, and furnished a report to the defendant, from which the printed statement was made. The latter was substantially true. He informed the defendant on his word of honour that the report was true, and rather an under than an over statement of the facts. The witness then described the circumstances attending the operation in nearly the same words as the alleged libel. After which he proceeded as follows.—"I examined the parts after they were removed from the body, in the demonstrating room. Several of the pupils were present. My attention was principally directed to ascertain the cause of the difficulties which attended the operation. I found the prostate gland slightly enlarged, and on the left side a smallish oblique cut. The parts around the gland appeared to have been bruised, and were dark-coloured. On the under part of the neck of the bladder there was a little projection, about the size of the tip of my little finger. This I took to be an enlargement of what is called the third lobe of the prostate gland. I found, on passing my finger on the under part of the bladder, that it passed up between the bladder and the rectum with the greatest facility. I did not make use of the slightest force in doing this, nor did I break down any structure. I was going on with my examination, when Dr. Hodgkin came to me very angrily, and said, "I wish people would not come here who have no business, and pull things about." He also said that somebody had broken down the fungous growth which I have described. I was conscious that I had not touched it, and I assured him that I had used no violence whatever in examining the preparation. I do not remember the name of any other pupil who was present but that of Mr. Pearl. There were six pupils present. Dr. Hodgkin said it was not a deep perineum. I think his expression was, "There is nothing remarkable about it." Mr. Key said it was not a deep perineum. I said to Mr. Key, "It seems to me the staff which you invented will never do for a deep perineum." Mr. Key replied that this "was not a deep perineum, and that if I thought so, I knew nothing about it." I saw nothing in any part of the preparation to account for the delay in the extraction of the stone. *I never saw a perineum in which I was unable to reach the bladder with the finger.* My impression was, that in the case in question the bladder could have been reached with the finger. I did not see in the neck of the bladder, or the prostate gland, an incision like the form of the gorget which was used in the operation. If the gorget did not pass into the bladder, it was likely to pass between it and the rectum. I have never seen the gorget passed between the bladder and the rectum by a skilful operator, or by a person having any pretensions to skill. The gorget was used subsequently to both the knives. The knives were not used more than once each. I cannot speak to the cutting gorget being introduced more than once. I never saw an operation performed in a similar manner. The operator did not appear to be in a state of self-possession. I do not believe the patient could recover under such an operation. I do not think Mr. Cooper a good operator, *but I once saw him tie the subclavian artery in a very skilful manner. That is not a difficult operation to a man who has any nerve.* I do not think Mr. Cooper's abilities are adequate to the office of surgeon to Guy's Hospital.

Cross-examined.—I am a surgeon, and have been in the profession 13 years. *I consider myself more competent than Mr. Cooper.* I am 28 years old. About six years ago I began to attend the hospital, and was admitted a surgeon three years ago. I am now practising. I have contributed largely to the *Lancet*, and derived a considerable emolument from it. (After some hesitation) I did not derive more profit from this source than from my profession. I received eight guineas per month for a certain quantity, and extra payment for all beyond that quantity. The payment of the extra quantity was in proportion to its length. The work is said to be clever, but does *not indulge in personal attack*, except in the cases of public functionaries. I cannot say that the circulation of the work is increased by these attacks. The publication produces the defendant a handsome income. I do not remember that Mr. Cooper ever threatened to turn me out of the room. (Question repeated, and after considerable hesitation)—I do not remember that Mr. Cooper threatened to turn me out of the room; but some angry altercation took place between us, and *I left the room.* I do not *believe* that I was turned out. This was at a public dinner. I never remember on any other occasion Mr. Cooper saying "Either you or I must leave the room, unless you make an apology." The surgeons of the hospital are nicknamed *bats*. Some altercation took place between us respecting my use of that word. I told Mr. Cooper that I did not mean to apply it personally to him. I do not remember ever to have said,—"I will watch my opportunity and make him repent it." *I will not swear that I did not do so, as I am a man of warm feeling, and say many things which I do not mean;* but I do not think it possible that I could have said so. The report of the operation was part of my weekly contribution to the *Lancet*. There were one or two expressions in the manuscript more severe than what appeared in print. I had stated that the operation lasted more than an hour, and the defendant said he would rather be under than over the mark. I have not seen the manuscript since the publication. I saw no gush of urine during the operation, and that I think a reason for supposing that the knife *never entered the bladder*. I have some doubt whether Mr. Cooper said, "Bring me my uncle's knife," or "bring me Sir Astley's knife." I have taken the examination of the defendant's witnesses, and from that arose my doubt. I put the report in the dramatic form, and furnished the quotation from Bell. *I intended to convey the impression that the patient had lost his life from want of skill.*" I did not think it a subject for joking on. I appended a note, in which it is represented that the man came to town to be "operated on by the nevey of the great Sir Arstley." Mr. Hodson, of Lewes, sent the man to the hospital. I know Mr. Clapham, a licentiate to the Apothecaries' Company. He is my cousin. *I did not assist in procuring his licence.* I was surprised at his getting it, as he was not of age. I have not made any bets on the issue of this cause. I have said that the odds were so and so. I do not recollect what I said the odds were. I have taken pains to collect evidence. I do not know that the witnesses have been shown a model, and heard a lecture to prove to them that the forceps passed between the bladder and the rectum. A lecture was given by Mr. Grainger. I do not know whether the lecture was given with a view to this trial; *but I myself have demonstrated the parts to persons who were to be witnesses in the cause.* I examined the parts with a view of refreshing my own memory. The defendant was there whilst I was explaining the parts to persons who were to be witnesses. The defendant also explained them to the same persons. I will swear that I did not hear the defendant say, Mr. Cooper "murdered the man as much as if he cut his throat with a knife." *I will not swear that I did not make use of the expression myself. I have heard explanations given to persons who were to be witnesses in this cause four or five times within the last six weeks.* I stood very close to Mr. Cooper during the operation, on the left hand side. I do not recollect that Mr. Key said he had used the straight staff, in cases of perineum, twice as deep as the one in question. I remember he told me that I knew nothing about it. I have been refused admission to Guy's Hospital since the publication of the report. *I was turned out of Middlesex Hospital four years ago*, and I have also been refused admission to St. Thomas's on account of the report.

Re-examined.—I was *expelled* from Middlesex Hospital because I was connected with the *Lancet*. I never sent a single report from that hospital before I was expelled. When I spoke of the odds respecting the cause, I meant, that as we had a great deal of good evidence, I thought the chances were much in our favour. I have not offered any bet. Mr. Grainger's School of Anatomy is of high repute. The lectures were open to any person. Several of Mr. Grainger's pupils were subpœnaed on each side. [*This witness gave his evidence during the cross-examination with evident reluctance, and towards its close the Lord Chief Justice reproved him, observing that he had not "answered one question in a straightforward manner like a man."*]

Alexander Lee was the next witness, and deposed as follows:—I am not acquainted with Mr. Cooper. *I never spoke to him.* I have been five years in practice as a surgeon. I saw Mr. Cooper perform the operation at Guy's Hospital. *It was performed in the usual manner, but was tedious.* It was the first time I ever saw Mr. Cooper perform the operation of lithotomy. I have seen the operation performed from 50 to 100 times. It is usually performed in ten minutes. I only know one instance of its lasting a quarter of an hour. I do not know any circumstances which occasioned the delay in Mr. Cooper's operation. Three instruments were introduced. I did *not observe whether the operator was collected.* I am not sure whether the forceps was introduced into the bladder on the first attempt. Mr. Cooper re-introduced the knife to make a second incision. The operation lasted for more than half an hour. I think it did not last an hour. I have no hesitation in saying, that it lasted from half an hour to 40 minutes. I am not prepared to give an opinion as to *whether the operation was skilfully performed.* I consider Mr. Callaway a better surgeon than Mr. Cooper. Generally speaking, the report in the *Lancet* is correct. I consider the form of the report objectionable. *Some expressions in the Lancet I did not hear the operator use.* The stone extracted was small.

Cross-examined.—I have been a merchant's clerk, but was bred a surgeon. *I dealt in potatoes about* 10 *or* 12 *years ago.* I have operated in lithotomy on dead subjects. When an operation is in hand, *no person can so well explain the difficulties as the operator.* It often happens that what appears ambiguous to a by-stander, the operator, if asked, would be able to explain. This is more particularly true where the operation is performed by feeling only, and not by the eye. *It is rash to give an opinion of an operation of this nature without asking the operator to explain what appears doubtful. No surgeon of experience would venture to give an opinion without speaking to the operator. I think it most presumptuous and rash in a young man and a pupil to give an opinion without speaking to the operator. Next to the operator the person most competent to give an opinion is the assistant-surgeon. The report in the Lancet is a very unprofessional report.* The mode of operating for the stone is not settled in any country, and any surgeon uses what instruments he pleases. I saw a small discharge from the first incision. It was impossible to say whether it was blood or blood and urine mixed. Sometimes the stone is bedded in the folds of the bladder, which contracts on the approach of an instrument. On these occasions it is better to allow the instrument to remain in some time.

Re-examined.—I think Mr. Cooper owed it to the class to give some explanation of the cause of the unusual difficulty. It is possible that a skilful operator would have discovered the cause of the difficulty.

Thomas Bolton.—I am a surgeon. I read the report in the *Lancet.* It is generally correct. The operation lasted an hour. I never saw so many instruments employed before. The operator was not in a state of self-possession at first, but he recovered. I never saw the cutting gorget used at the same time with knives on any other occasion of a similar nature. Before Mr. Cooper extracted the stone, he said he could not explain the cause of the difficulty. I do not consider that the operation was scientifically performed.

Cross-examined.—The operation in question was the sixth I have seen performed. I have seen none since.

Benjamin Harrison.—I am treasurer to Guy's Hospital. I have held the office 31 years. Mr. Cooper was elected assistant-surgeon on the 14th of May, 1825, and Sir A. Cooper was elected to the office of consulting-surgeon on the same day. When Mr. Cooper was elected, he was considered perfectly competent to the office. None of the governors of the hospital are surgeons; but they have daily opportunities of ascertaining the qualifications of the apprentices. *Mr. Cooper was elected to his office because he was best fitted to fill it. He would have been elected if he had not been Sir Astley Cooper's nephew.*

The defendant pressed the witness to state whether he did not consider Mr. Callaway a better surgeon than Mr. Cooper. The witness said he did not like to give an opinion upon so delicate a point.

The Lord Chief Justice said, that the question was *a very invidious one.* Supposing the merits of the two gentlemen to be equal, the governors had a right to elect Mr. Cooper if they thought proper.

Cross-examined.—Mr. Cooper was demonstrator under his uncle, and *gave great satisfaction.* He was recommended by all the surgeons in the hospital. Sir A. Cooper did not know that the hospital intended to elect his nephew, till I informed him of it. I knew that Mr. Cooper had served in Norwich Hospital, and also as army-surgeon in Spain, under the Duke of Wellington. He likewise served in the same capacity in Canada, at the close of the last American war. He afterwards studied at Edinburgh for two years. He then came to Guy's Hospital. Mr. Cooper has always maintained the reputation which induced the hospital to elect him.

Mr. Wakley then proposed to put in the preparations taken at the hospital, as part of his case, stating at the same time that an adequate examination of them could not take place in the glass.

Sir James Scarlett said that if the defendant could not make out a case without these preparations, he could not make it out with them.

The preparations were then brought into court, and were examined by several medical gentlemen.

Lord Tenterden, (addressing the defendant,)—Whom do you call to speak as to those preparations?

Mr. Wakley.—I call Mr. Holdiman Partridge.

Mr. Holdiman Partridge examined.—I

have examined these preparations. Whilst they are in the glass I cannot see the incisions; I see the opening in the bladder. I cannot give any reason why the operation should have lasted an hour, without having the preparations in my hand; and I would not like to give a decisive opinion on the subject without having examined them by myself. I cannot, as the preparations are now before me, say whether the incisions are oblique or horizontal.

Mr. Wakley.—My Lord, I have not had an opportunity of examining the preparations, nor have any of my witnesses.

Lord Tenterden.—I can't help that, Sir.

Mr. Wakley said that his case was now closed.

A short conversation took place between Sir J. Scarlett and the Lord Chief Justice, as to the propriety of proceeding with the plaintiff's case that evening.

His Lordship seemed desirous that Sir J. Scarlett should open his case that night, and proceed with the examination of his witnesses to-morrow; but

Sir J. Scarlett submitted that it would be more convenient to the interests of justice that he should address his remarks to the jury when they were fresh, and not in such a state of exhaustion as they must be in at present, after the fatigue of the day. As far as his own personal convenience was concerned he would rather proceed that evening, whilst the facts were fresh in his memory; but as it was impossible to close the plaintiff's case that night, it would be more conducive to the interests of justice that it should be placed at once before the view of the jury.

Lord Tenterden reminded Sir J. Scarlett that to-morrow was Saturday.

Sir J. Scarlett was aware of it. It would not, however, make any difference, as he should not take up any thing like the time that had been consumed by the defendant. He should certainly have to call witnesses to support his statement. He should call some of the most eminent surgeons in London. He should not call many of them, but some of them he must call.

Lord Tenterden then adjourned the court till half-past nine o'clock to-morrow morning.

### SECOND DAY.

The hall-doors were thronged at an early hour by persons anxious to obtain admission into the court. The officers, however, adopting a different course from that of Friday, kept the crowd at the outer doors; and until the jury, counsel, and witnesses were admitted, those who had assembled merely from curiosity were not allowed to force their entrance. The number of eminent surgeons was equally great as on Friday. Among them we observed Sir Astley Cooper, Doctors Babington, sen., Addington, Bright, and Johnson; Messrs. Green, Key, Callaway, Travers, Tyrrell, and Brodie. The gallery and back benches were filled by witnesses and medical students interested in the cause.

As soon as the jury had assembled, one of them asked permission to put a few questions to the witness Lambert, who was examined on Friday.

Lambert was then called into the box, and in answer to questions put by the juryman, said, that he could account for the difficulty in the first introduction of the forceps into the bladder in only two ways—either that a very small incision had been made, or that the knife had not entered the bladder at all. The *post mortem* examination was made after the report had appeared in the *Lancet*. He did not think the lump would have made any difference, if the cutting had been made in the proper and usual manner.

Juror.—Mr. Lambert stated that there was a small projection on the inside of the bladder, which appeared to him to be a little larger than the tip of his little finger. Now, I want to know, whether this unusual projection or enlargement could not by possibility have occasioned the first difficulty in introducing the forceps?

Witness.—Certainly not, provided that the cut had been made in the usual manner.

Juror.—Might not this projection have occasioned the trickling, rather than the gush out of the bladder when the cut was made?

Witness.—I think not.

Sir J. Scarlett wished to know whether the witness had communicated to *The Times* and another paper any report of the operation on or before the 29th of March last.

Witness.—Certainly not; nor do I know how the notices got to those papers.

Sir J. Scarlett wished to ask whether the witness was aware that a notice did come out in those newspapers respecting this operation?

Witness.—I saw it in *The Times*; but I did not see it in any other.

The defendant wished to ask the witness whether he had the slightest reason to believe that those notices were transmitted through him.

Lord Tenterden.—We can't inquire as to the witness's belief. He may be asked as to his knowledge.

The witness stated that he had no knowledge on the subject one way or the other.

Sir J. SCARLETT then addressed the jury for the plaintiff. The time had at length arrived when the plaintiff was entitled to lay before the jury the grounds on which he sought redress, for one of the most injurious attacks upon his fortune and fame that ever appeared in a court of justice—invented by malice, and defended by falsehood. Hitherto he had been put on his defence, though he was the party complaining; and though he sought redress at the hands of a jury, such was the fate of human affairs, that during an entire day he had been placed upon his defence as if he had been indicted for a criminal offence; and up to the present hour the jury had no means of knowing of what he complained, unless, perchance, they had read the libel in the evening papers of Friday—furnished, no doubt, by the defendant, or his attorney, for the purpose of giving to that proceeding of which the plaintiff complained the widest possible circulation, without any antidote or explanation; thus striking still deeper into his bosom the injury he had before received. The proceedings of to-day and yesterday would furnish every reasonable man with grounds for grave and serious reflection. It was not for him (the learned counsel) to complain of the forms of law, or of the practice of courts of justice. He submitted, as every subject of the realm was bound to do, to the rules laid down by the court; but in a particular case he might perhaps be at liberty to suggest how unfortunate it was for an individual attacked by a gross and malicious libel that he should have the public interest excited to hear the accusation against him, and when that had subsided he was to be put on his defence. He (the learned counsel) used the word accusation, because he had no doubt that the jury all felt that they were now sitting in judgment, not upon the question what reparation should be made to an injured individual for one of the basest and most malignant libels which any man could complain of, but whether the plaintiff was not a person unworthy of the public situation which he filled.—a person who had contributed to shorten the life of a fellow-creature, and who was deficient of that skill and knowledge of his profession which he ought to possess, and which no man, having any skill or knowledge himself, had ever dared to doubt he did possess. He (Sir James Scarlett) had some reason to regret the fate that had attended his client—that when he entered upon the threshold of the court, and before he had time to procure the aid of the law, he met the sword of the assassin; and before he could obtain redress, the wound was made deeper and deeper into his side. He was traduced, and his character dissected, in the manner the jury had heard yesterday; and now the jury came with minds, no doubt, prepared to hear what it was he complained of, and on what it was that they were called upon to give a verdict. He (Sir J. Scarlett) was no enemy to the periodical press—far from it—though he had never flattered it, and never would; but he would say, that the example of this proceeding would give it a triumph and an influence which it never had before. He would now enter upon his case, and endeavour to dispel the doubts, if any doubts they (the jury) entertained, whether the plaintiff, Mr. Cooper, was such a person as yesterday he was represented to be. He was a gentleman who had the honour of being connected with Sir Astley Cooper, his (Sir J. Scarlett's) highly-esteemed and excellent friend. In early life, as soon as his profession was finally chosen, Mr. Bransby Cooper became a pupil at the Norwich Hospital,—the most distinguished, with the exception of those in London, for the operation of lithotomy. He served with diligence in that hospital for nearly two years. He then came to London, and was admitted a pupil of Guy's Hospital, where he continued for a year and a half, or nearly two years, when—as he (Sir James Scarlett) hoped he might be allowed to say, without offending any body by saying it—his merits, as well as his manners, recommended him to that notice by which he was appointed assistant surgeon to a regiment of artillery, which regiment he accompanied to Portugal in the year 1813, and was present in every battle till that of Toulouse,—that grand effort which crowned all our victories, and was the basis of the peace in 1814. His first exhibition of coolness was in operating in the field of battle, amidst the roar of cannon, and exposed to danger. His experience as a surgeon was known in that extensive field where a man required both confidence and talent, and he had the opportunity of shewing that talent which was the ground of his honourable relation's wishing him to pursue that profession for which his abilities appeared to render him so peculiarly eligible, and in which, if he pursued it with the interest and talent he had exhibited, he had a prospect of attaining great eminence. He went with his regiment to Canada, and served nearly a year in that unhappy war, in which we had the misfortune to engage with our friends in America. On its termination, and when he returned to England, he was sent to Edinburgh, and was admitted there as a medical student; and there he received all the education which a man could receive as a pupil. He was placed in the high situation of president of a society until he quitted it. He had it open to him to choose what line he thought fit. He might have taken his degree, and established himself as a physician; he had the example of his illustrious uncle in his view—one of the most distinguished as well as the most prosperous men in his profession. Sir Astley Cooper was the surgeon of Guy's Hospital; he had been the pupil of Mr. Cline, a man educated in the same school; Mr. Bransby Cooper became bound an apprentice to his uncle in 1817, and during that period his assiduity was unremitting. He (Sir James Scarlett) had a right to say so, from the evidence of that excellent and honourable man, who gave his testimony yesterday (who was intended to be insulted by the defendant), and who stated that his conduct was honourable to himself and satisfactory to all around him. Sir Astley Cooper (who made his nephew his demonstrator of anatomy) gave distinguished lectures there, and found him a valuable assistant. This led him to obtain an intimate knowledge of all the most abstruse parts of the profession, and he had an opportunity, which every man did not possess, to become by and by of the same fame and the same success as his honourable relative. He did more than that. Sir Astley, whose practice had probably been more extensive, for a number of years, than any other surgeon in the world, called by all sorts of persons to perform the most difficult operations, and who never, as those who knew him could say, allowed even the calls of the poor to be disregarded,—who bestowed as much in humanity as for gain—Sir Astley having daily and nightly requisitions, was obliged to do that which every person in his extensive practice must do—namely, have a person to assist him when he was called upon in a case of emergency, in order that his patients might not want the aid of a competent person. Mr. Bransby Cooper having received instruction in the same school as himself, Sir Astley found in him one of the fittest persons to assist him, and was able, according to the statements of that eminent man, to discharge the most important duties, when Sir Astley himself, from the impossibility of being in two or three places at the same time, was obliged to employ an assistant. It could not be supposed that such an employment was the result of favour. The surgeon who employed an assistant for that purpose, was obliged for his own honour and interest to employ a competent one; for the jury would consider what situation Sir Astley would be placed in, if on being sent for to perform a difficult operation, and he being at the same time otherwise engaged, he had sent for his substitute a person who was found to be incompetent, or suspected to be so. Sir Astley would in such a case have been ruined in practice as well as in reputation. He (Sir J. Scarlett) had therefore a right to say, that not by his education only, but by that best testimony which Sir Astley bore to his nephew's fitness and capacity, that that fitness and capacity were established beyond all doubt on the most substantial evidence. His apprenticeship expired in the year 1823. He had at that time considerable experience and great practice. He then became a surgeon on his own account, still, however, continuing to render assistance when his uncle required it. And now what happened at Guy's Hospital? That establishment,—about which, for the present, he (Sir James Scarlett) should say nothing, except that it was highly useful and of the greatest advantage to the poor,—that establishment, or rather the individuals connected with it, and the Governors, upon whose character no impeachment was ever made until the scandalous publication appeared, thought it expedient, for the advantage of their charity, to establish a school of anatomy as well as that which existed at St. Thomas's. They had a right to do so, and having done so, who was it that dared, unless he defied all decency and common sense, to complain of their conduct because they sought for a surgeon in their own resources? Unless because they thought in their own hospital, with the concurrence of eminent surgeons, it would be well to found this school, who was it that dared to complain that they did not advertise in the newspapers and seek for information from the *Lancet?* The Lamberts and the Wakleys would then have been ready to come forward from their dark recesses, to have rendered their assistance in choosing surgeons for the hospital. It was not from the school of Cline, Astley Cooper, or Green, no, nor even of Mr. Callaway himself, that surgeons ought to be elected; no, it was from the recommendations and patronage of the editor of the *Lancet*, Mr. Wakley, that information was to be obtained on such a point. That was the wound which had sunk deep into the defendant's breast; his dignity as the editor of the *Lancet* had been slighted, his knowledge of surgery had not been appreciated, and the immense circulation of his work had been disregarded and passed unnoticed by the governors of Guy's Hospital, who had thought right to elect out of their own body an individual whom they considered in every respect competent to fill the situation of surgeon. He hoped the jury would think that the governors of the hospital did not deserve to be tried and executed, because they thought it right to look into the school of Cline and Sir Astley Cooper for a surgeon to supply the vacancy in the hospital. But the defendant had attempted to prove that which he had the audacity to allege—namely, that it was through the instrumentality of Sir Astley that Mr. Bransby Cooper was placed in that situation, and that he was placed there without regard to his professional merits. He had not however proved that fact, but had proved the very reverse. He (Sir James Scarlett) had now stated the history of Mr. Bransby Cooper, and the circumstances under which he had been appointed one of the surgeons of Guy's Hospital. It was now time that the jury should hear who the defendant was. He (Sir James Scarlett) knew nothing of him, nor should he have known him except for the occurrences of yesterday; but he could not help observing his extreme ignorance in the art which he professed. He was as ignorant of his own profession as he was of good taste. But the learned counsel spoke of him only from his exhibition of yesterday; he had never heard of any operations performed by him, and he believed he was known to the public exclusively for publishing the *Lancet*, which it appeared he had projected himself. That work, it should seem from his own statement, was established for the purpose of publishing lectures delivered at the hospitals,—in other words, for the purpose of committing plunder on the property of others to assist himself. What! was it to be said, that if Mr. Cline or Sir Astley Cooper, or any other eminent surgeon, should compile a course of lectures, and deliver them to the pupils of his own class at the hospital, who paid him for attendance, and remunerated him for those labours, that a periodical paper should rob him of all advantage, and, without his leave and licence, make them public, so as to give to all the pupils in the kingdom who were desirous of studying his art, the advantages which the lecturer thought he had established for himself;—that he should rob and injure him, and gain ten times more than he did himself acquire, after being at all the labour of compiling his lectures; that he should do that which would render it unnecessary for the pupils to attend the lectures, because all the advantages derivable from their attendance might be gained by reading the reports of them in the *Lancet?* Could it be supposed that there were any persons in the honourable profession to which Sir Astley Cooper belonged, who were so base and ungentlemanly as to make use of the privilege which was allowed them of attending these lectures, for the purpose afterwards of giving them to the world without the leave of the lecturer himself? Yes, there were those who were contributors to the *Lancet* who were base enough to do this, and who thus enabled the editor (Mr. Wakley) to make his five or six thousand a year, and to gain the reputation of being a "popular writer!" The defendant had himself avowed that the *Lancet* was a work founded on the principles of robbery and plunder. He stated that he obtained his communications from pupils at the hospital, and he called them "men of honour!" —men who were induced to betray their trust and surrender their honour, and who, by making contributions of the lectures they heard, furnished that to the public which ought never to come out of the walls of the hospital except by the consent of the lecturers themselves. But this was not a robbery of property merely—it was a robbery of character and reputation. He (Sir James Scarlett) was glad that he was addressing gentlemen of education, as he only wished that this matter should be judged rightly. He would ask whether, supposing one of them had taken great pains to prepare a course of lectures which by-and-by he intended to publish himself, could he endure it if an unfledged pupil who had been admitted to the hospital and allowed to take notes for his own instruction was afterwards to furnish them to a person who meant to commit them to the press without those revisions and corrections which an author was generally anxious to make in his works before they were submitted to the public eye? Could any man, and particularly a public lecturer, suffer such use to be made of his works? Suppose one of the jury trusted to a person the key of his cabinet, which contained written communications, and that he made extracts from them and sent them for publication in the *Lancet?* Would they not think that that man was one of the basest of his kind, and would they allow him to enter their doors again? And yet these were the persons who contributed to the *Lancet*, and these were the means by which the editor, Mr. Wakley, was enabled to roll in his carriage, and laugh at the parties whom he thus robbed and plundered. He (Sir J. Scarlett) was, upon the defendant's own confession, justified in saying that this work (the *Lancet)* was a sort of literary raven, which lived by plunder and shamelessly held up its head by the injury which it inflicted on others. He (the learned counsel) now came to consider what had been done by the plaintiff, Mr. Bransby Cooper; and the jury were not to believe that they had had any information on that subject from the witnesses whom the defendant had examined yesterday—from such persons as Mr. Lambert, the reporter, the pupil of six months, or the demonstrator of Mr. Sleigh's school, situate at No. 1, Dean-street, in the Borough!—they must not suppose that they had had any information whatever on the subject from those persons. The defendant had called but two witnesses whose evidence was deserving of the least weight; one was Mr. Holdiman Partridge, who was a surgeon, and the other, Mr. Lee, who was a surgeon, and also a dealer in potatoes. The acquaintance between the editor of the *Lancet* and Mr. Partridge had no doubt very rapidly improved. He (Sir J. Scarlett) saw them sitting together, and no doubt whatever praise should be due from the *Lancet* to Mr. Partridge he would have it. It would, however, not be any praise which the respectable part of the profession would value coming from such a source. A critical work like the *Lancet*, which mixed a great deal of vulgar ribaldry with its reports of cases of surgery,—ribaldry which by some people was called wit, and was perhaps acceptable to the mass of persons whose tastes were not very refined—a work of this sort would, no doubt, have some weight with the vulgar; but by people of learning and of honour it was looked upon with contempt. Every person who contributed to, or patronized, the *Lancet*, was celebrated in it. Every one who did not was abused by it, and no eminence, no skill, could protect a man against the attacks of the Lamberts, the Wakleys, and the young pupils of six months, who would be lifted up in superiority over the pupils of the Coopers, the Traverses, and Brodies, and all others possessing the feelings of gentlemen and of men of honour. "Gentlemen," continued the learned counsel, "there is a certain privilege which belongs to this high profession—it is something that is better felt than described: and the man that does not feel it will not be capable of appreciating the argument I address to you. In the ordinary traffic of life, called commerce, there is a course of plain dealing, of simple integrity, that marks the line between honesty and the mere appearance of it; and the meaning of it is well understood by every man. But in the practice of a liberal profession there is a certain feeling of honour which becomes a gentleman, and which a gentleman only can feel; which renders it not sordid, but which gives it a character which belongs to such a profession—a certain dignity—a certain pride which makes a man feel that profit is a secondary object to him —that fame and reputation, and means of utility, are its greatest recommendations. Either in the profession of the law, or the profession equally honourable, and perhaps equally useful, or more so, that principle is debased, that principle is destroyed, if a man finds that it depends upon whether he makes concessions or no to the editor of such a publication as the *Lancet*. If he find that it depends upon whether he makes useful contributions—that he should court the author, to acquire that fame which before was acquired by honourable competition and fair means, it introduces into the profession a means of degradation that dispossesses liberty, and finally destroys it. What would you say, now, of the different situation of surgeons, if at this moment any of the honourable persons in that situation were to tell you that all their reputation was acquired by such means, and that a man, if he has the misfortune to offend Mr. Wakley, the editor, or some contributor to the *Lancet*, although he may have that right feeling and those refined sentiments which would fit him for the honourable profession of a surgeon, yet still, having offended this person, he is to be held forward to the public as unworthy of the situation which he holds? What, if in the law,—as in every place where persons are employed in doing that which enables them to publish their peculiar efforts,—if they were not to allow that fair competition in a Court of Justice to determine who is the advocate which the public should employ, but that it should be given beforehand by suppression and false representation, would not that destroy the honour of the bar; and would it not tend to degrade still more that honourable profession, if among themselves persons were to be found capable of making reports of their own exhibitions? Such a thing cannot be found in the profession to which I belong. But it has an example in the profession of which we are treating to-day, for you have Mr. Lambert, who is said to be a surgeon, getting his eight guineas a month, (and depend upon it Mr. Lambert's fame will depend much more upon the fame he gets in that publication than he will get from any honourable member of that profession,) he it is who furnishes contributions from the hospital, where he has the privilege of attending. Such a course it is that degrades a profession—such a course it is, that from my very heart and soul I detest and abhor." But to return to the immediate subject of the operation which Mr. Bransby Cooper performed at Guy's Hospital. Mr. Cooper was now of the age of 34 or 35. He had performed many operations for the stone—many at Guy's Hospital—with complete success, but those successful operations had not been reported by the *Lancet*. He had also performed many other operations of the most difficult and complicated kind. There was a time when this operation of lithotomy was attended with almost certain loss of life. The improved state of surgery, however, had rendered the consequences of this disorder less dangerous, and the number of those who died, in comparison with those who were saved, was very few to what it formerly was. It was generally considered now that the number dying was about two in fifteen—that is, one to seven and a half. Formerly, they very rarely escaped. At one time the numbers were as one to four or five; now it was one to seven and a half. The disorder sometimes attacked infants, and in that form it was most easily dealt with. In persons of that tender age the stone might be extracted almost to a certainty without hazard. With others the danger increased; but Mr. Bransby Cooper had performed the operation on perhaps one of the oldest men that ever suffered it—a person whose age was 87. The operation was never performed on an adult till he himself felt that the pain he suffered or the apprehension of the loss of life was greater than the risk of the operation. Every man was to judge of that by his own feelings. There was something in the apparatus more terrible than in the operation itself—something that operated on the mind against being the subject of it; and it was nothing but the extreme pain, or the apprehension of loss of life, or under the hope of being relieved from it, that gave a party courage to submit to it. The jury might suppose, therefore, that no surgeon was ever called upon to operate unless under extremity, where the patient says—"I cannot live, I must die under it. The urgency is so great, that I call upon you for instant relief. All judgment is at an end. You must perform the operation, or I must die." What course was left but to perform the operation? Now the jury would understand that the particular practice of different surgeons in some slight degree varied; but they were all employed for the same end. It was not for him (the learned counsel) here to describe and remark upon the merits of the different schools. The jury had heard that the French hardly used any of the instruments that we did. We used the instruments that we thought best, and those which were calculated to give the least pain and uneasiness to the patient. The learned counsel then went on to describe the mode of the operation. The first operation was to ascertain the existence of the stone. That was done by introducing through the urethra a sound—a small rod of steel; which being introduced through the tender passage found its way into the bladder. Then by turning it about a little, if it encountered a hard substance, striking it with the instrument, the operator heard the sound of it, and ascertained the existence of stone. That being done, then came the operation. For the purpose of the operation the patient was tied down in the simplest manner, an incision was then made in the perineum. The finger was introduced into the bladder, and it very often happened that when it was, the stone was thrown out in a moment without any other instrument. In children the parts to be operated upon did not lie far from the surface, and were easily reached by the finger; but in proportion as the person became adult, the parts swelled, and therefore it was necessary that instruments should be had recourse to. No man should ever attempt to attend an operation of this kind without having a number of instruments with him. He would be most presumptuous if he did. The eye of the operator did not enable him to see in what situation the stone was placed; it was all touch and feel; consequently a variety of instruments were necessary; and every man who attended as a surgeon knew that he was bound to have all the instruments that were used in the school of surgery in which he had been taught. The first operation was, then, to introduce again into the urethra the sound or staff. Mr. Key, a most eminent surgeon of the hospital, had been himself the inventor of the staff. He considered it highly useful; by some it was used, by others not; that was mere matter of opinion. What was called the staff was a straight stick, not curved, with a point. The urethra was a long canal, which passed through what was called the prostate gland, before it entered the bladder. In the prostate gland it had a communication with other parts of animal life, but it was opened into the bladder. On passing the staff the opening into the prostate gland was penetrated, and it was inserted into the bladder. The staff had a curve in it which was passed in front, and between the legs of the patient; and the use of the curve was, that when the operator introduced his knife it touched the point of the curve. When the point of the knife was in the staff, he then knew that he was in the true direction of the urethra—that he there could get the prostate gland, and by bringing the staff forward, and altering its position a little, he got into the bladder. If the forceps could not find the stone, then the sound was introduced through the opening, and it was a common practice, if the stone could not be felt, to try sounds of different forms. The stone might be enfolded in the bladder, or so suspended that it might be extremely difficult to get at it. Then the operator had recourse to the scoop, which was an instrument like a tea-spoon in its shape. With this an attempt was made to touch the stone, and thereby cause it to fall, so that it might be got at by the forceps. Now, it sometimes happened that there were cases in which the most skilful operator was baffled in his exertions to reach the stone, or to find out the position of it, and where the cause why its situation could not be ascertained had been only discovered upon a *post mortem* examination. A surgeon might be convinced that the stone was lodged somewhere, but still in a position where he could not reach it by ordinary means. In that case, other means were resorted to, and a second incision became necessary. This was the usual course of the operation, but he would come now to the operation in question, which was performed by Mr. Bransby Cooper. The patient had been sent up from a parish in Sussex. Now, there were eminent surgeons at Brighton, and it was therefore only fair to assume that the case was one of great difficulty, or the parish would not have gone to the expense of sending him to London to be operated upon. However, he was sent to the hospital and Mr. Bransby Cooper saw him. Although he was a short man, with a hectic complexion, Mr. Bransby Cooper ascertained that his kidneys were in a disordered state, and that it was necessary to postpone the operation until this disorder was abated. It was accordingly postponed, until the agony he suffered rendered the operation no longer proper to be delayed—until, in short, it appeared that it must be performed or the patient must die. Mr. Cooper made an incision, and there was an immediate flow of urine—not a gush; for under such circumstances no gush could ever take place, owing to the collapsing of the parts. He then inserted his finger, but could only reach the prostate gland, and could not reach the bladder. This he spoke of at the time. Feeling, however, by his finger in the prostate gland, that the wound was in the right direction, he put the forceps in to see if he had reached the bladder. This was most easily to be ascertained by the forceps, for the forceps being shaped like a pair of scissors, could easily be expanded within the bladder; and if the stone was in the ordinary situation, he would easily lay hold of it; but the forceps could not be expanded in a solid substance like the prostate gland. Mr. Cooper, however, could not find the stone; he had no idea even where it was. The first notion was, that the previous indications were fallacious, and that there was no stone at all, and Mr. Cooper felt very anxious, as any person would naturally be under such circumstances. He then had the option to carry his instruments all round the bladder, to feel for the stone, which would have required force, or make a second incision; he chose the latter, and, calling for Sir Astley Cooper's knife, he enlarged the wound, and then again tried the forceps, but without success. He then thought he would ascertain with the sound if indeed there was a stone, and then, upon withdrawing the sound, the curved point of that instrument struck the stone. This proved that the stone lay, as it were, upon the upper part of the pubis, and accordingly it became necessary to resort to the bent forceps, which, by means of its curvature, might touch the stone and bring it down. The bent forceps, however, could not touch the stone; and what was then to be done? The only mode was to make a wound in the prostate gland, large enough for the forceps to be introduced through it. Mr Cooper then used the gorget, but not the blunt gorget. It was false to say that he had used the blunt gorget. He used the cutting gorget, which was like a prolonged scoop, and which had two advantages—first, that of making the wound sufficiently large; and, secondly the advantage of not being able to make it too large, for it could not make a wound beyond a specific size. According to all the rules of science, the cutting gorget would make a wound large enough to admit the straight forceps, which, while the abdomen was pressed down, would catch the stone. This was the course adopted, and it succeeded. It was perfectly true that Mr. Cooper did say that he could not imagine what was the difficulty of the case, but when he had discovered the difficulty, he had used the most skilful and the most prompt means of overcoming it. The stone was flat, and lay as it were upon the shelf of the pubis. There was no protruding end for the forceps to lay hold of; therefore the form of the stone and its position fully accounted for its not being caught by the forceps. What passed subsequently? That which always took place after such an operation, and which was due to science to be carried into effect—a *post mortem* examination and preservation of the parts. In the course of the operation Mr. Callaway had himself (and Mr. Callaway was admitted to be a man of skill) thrust his finger into the wound, to see if he could reach the bladder, but he could not do so. It was an utter falsehood to say that any force was used. He would call Mr. Callaway, who would tell them that it was a most gross and calumnious exaggeration to say that any force was used. On the opening the body, a gentleman who went to witness it for curiosity, put his finger into the wound, and could not reach the bladder, owing to the depth of the perineum. Dr. Hodgkin had not said that the perineum was not deep, though he had said that it was not deep in proportion to the size of the man. The wound in the bladder precisely corresponded with the external wound, except so far as a small slip of no importance in the prostate gland, had not hit the very spot of the original wound. There was a cellular membrane between the bladder and rectum, which was very easily broken; but no breach had taken place, and it was perfectly sound. The kidneys were diseased, and that alone might have contributed to the man's death without the operation having been performed at all. Dr. Hodgkin made an observation that this membrane was easily lacerable, and yet it was perfectly sound. As soon as Dr. Hodgkin turned his back, Lambert took up the part, and exclaimed, "There is an opening between the bladder and the rectum;" upon which Dr. Hodgkin, who is a Quaker, immediately replied, "If there be an opening, friend, it is thyself hast made it," *and he had made it*. Mr. Key would prove, and so would Dr. Hodgkin, that there was no opening when they had examined the parts immediately before. He should prove to them the stupid ignorance of Lambert, for if he had sense, he would know that if the opening was made by the forceps, it would have been traceable by the extravasated blood; but this was not the case—there was no extravasated blood. If such a wound had been made as had been described by Lambert, it would necessarily have produced death in a much shorter time than 48 hours. The whole of the statement respecting the nature of the wound was a gross and infamous calumny on the part of Lambert. The jury would, perhaps, ask who this learned man—this Mr. Lambert—was, that he should thus set himself up as the censor of the proceedings in the various medical schools of the metropolis?—who set himself up to canvass the conduct of the most eminent medical practitioners of the day? Why, this Mr. Lambert—this able and celebrated surgeon, according to his own statement—was engaged to report these cases and to make strictures on the conduct of his instructors in surgery for the *Lancet*, at a salary of eight guineas a month. But this Mr. Lambert was not only a distinguished surgeon, as he would wish them to infer, but he also set himself up for a wit. This worthy contributor to the *Lancet* endeavoured to make a dramatic sketch of the scene which he had described, and he had divided it into acts and scenes. The sufferings of the person who was undergoing a painful and difficult surgical operation were made the subject of a dramatic treat for the entertainment of the readers of the *Lancet*. He began his account with making some silly jokes and allusions to what he was pleased to call "uncle's knife," which were certainly below observation. The learned counsel then made some observations on the notices which had appeared in the *Lancet*, stating that the operation had been performed, and that they should describe it in a future number. This was the first announcement of the case—"My uncle's knife, and half a dozen other instruments.—*Post mortem* examination. On Tuesday last an operation took place by Mr. B. Cooper, and there were used Mr. Key's knife, the cutting gorget, my uncle's knife, the blunt gorget, &c.; and on Wednesday evening, as might be expected, the man died." Now what would the jury think of the feelings of the man who could witness the operation, as he said that it had been performed, and then endeavour to make a joke of it, and turn it into a drama for the amusement of the public. "I know not," said the learned counsel, "if the choice were given me, whether I should choose to be the inventor of such a calumny, gross and false as it is, or the reporter of it, if true, and yet capable of throwing it into such a shape as this." With respect to the conduct imputed to his client, he did not hesitate to say that such conduct as had been imputed to him could not have been displayed by any man, and he was sure that there was not one word of truth in the statement, as far as regarded Mr. Cooper. All the scene with respect to holding up the forceps over his head with an air of triumph, he was authorized to say, and he would prove it by evidence, was utterly without foundation. The learned counsel then proceeded to read the libel, and commented on it at great length. In this article it was intended to convey the idea that in the first instance Mr. Cooper did not make a sufficiently large incision, and that it did not even reach the bladder—that he had endeavoured to make use of the forceps before he had cut the bladder to admit of their use with any degree of service—that he had made a second incision in a highly improper situation—and that he throughout the whole of the operation had used a degree of violence which, instead of being essential to the performance of it, was only calculated to give pain to the patient; and also that the length of time which the performance of the operation had taken up was unnecessary, and had caused the death of the patient. The defendant had met the charge by boldly avowing that what he had published was true, and that Mr. Bransby Cooper was an unskilful practitioner, and unfit to be a surgeon at Guy's Hospital. One of his pleas was to this effect:—" I am editor of the *Lancet*, in which I publish reports of the cases which occur in the public hospitals; and I published the report in question as containing an account of an operation performed at Guy's Hospital." Now he (Sir J. Scarlett) did not know how Guy's Hospital could be considered a public one, as it was never supported by public contribution. It did not shut its doors against those who went there for the purposes of science, although it was anxious to exclude such as obtruded themselves for the purpose of inventing and disseminating calumnies. The defendant went on to say, " My reporter brought me, in the usual course, the reports of this case, with critical remarks, and I, as in duty bound, published it, believing it to be true, and not knowing to the contrary." So that if the editor of the *Lancet* got from a person to whom he gave eight guineas a month, a falsehood, however infamous, he was in duty bound to publish it to the world. This was one of the most extraordinary pleas which he (Sir James Scarlett) ever remembered to have seen. In two numbers of the *Lancet* which appeared subsequently to those which contained the libel, some observations were made on the circumstance of a patient at Guy's having left that hospital and gone to St. Thomas's. In No. 241 of the *Lancet* it was stated that no operation had been performed at Guy's Hospital during the last fortnight, and that since the operation of lithotomy performed by Mr. Bransby Cooper, a patient had become so alarmed, that he had " decamped," and gone to St. Thomas's Hospital, under the care of Mr. Travers. In the following number (242) some lines appeared in allusion to this statement. They ran thus—

" On the patient who decamped from Guy's Hospital.

" When Cooper's *nevey* cut for stone,
" His toils were long and heavy;
" His patient greater skill has shown,
" He soon cut Cooper's *nevey!*"

The jury, from this specimen, would be able to form some opinion of the wit and humour of the defendant, and his contributors to the *Lancet*; and they would also be able to judge of the motives by which he had been actuated in publishing the report of which Mr. Cooper complained. Bad as the libel was, and base as were the motives which gave it birth, the defendant had not produced a single witness who did not entitle the plaintiff to a verdict. All the witnesses in succession establish beyond the possibility of doubt the falsehood of the libel, because the pleas on the record were those by which the defendant must stand or fall. Those pleas could not be pretended to have supporters. Each witness did more than not establish the truth of the charge, for each contradicted the other. The first and most competent person to be placed in the witness-box was the author of the libel himself. That man was Lambert; it was admitted that he had upon two occasions had differences with Mr. Cooper. He was suspected of contributing to the *Lancet*. It now turned out that he was receiving eight guineas a month for his contributions, and more, according to the quantity of the matter which he supplied. At a dinner at Guy's Hospital he gave offence to the company, and was ordered by Mr. Cooper and others to leave the room. Efforts were made to extract that fact from Lambert upon his examination; at length he said he remembered that on finding his presence disagreeable, he left the room. Upon another occasion, a short time before the report which was the subject of the present trial, he was suspected, but not then known, to be a spy to the *Lancet*. It appeared that he had used the term " Bat," a word invented by the defendant to designate hospital surgeons, to make them ridiculous, and his work the more popular. Mr. Cooper was offended with the expression. When questioned upon this point, Lambert said " I certainly did not mean to give offence to Mr. Cooper." Did you not on your oath say that you would watch an opportunity of making him repent it? " I cannot swear that I did; I am a man of warm temper; I cannot swear that I said so; I remember being called on for an apology." Yes, he did watch an opportunity of destroying Mr. Cooper. He went to the man who gave him eight guineas,—a thing which no surgeon who has the slightest pretensions to respectability would think of accepting. "On my honour," says he to the defendant, "it is true." The defendant, however, lops off some part of the libel, before he inserts it. *Lambert puts his finger in between the bladder and the rectum, and then says Mr. Bransby Cooper had forced the forceps in that direction,—a statement which, unless he was ignorant beyond what could be supposed, he must himself have invented for the purpose of destroying the reputation of an individual, towards whom he had before vowed vengeance.* "In the whole course of my professional experience, (continued the learned Counsel,) I never saw a libel, the malice of which was more distinctly proved." The question then was, who was to bear the consequences of this publication. The defendant had come forward and boldly avowed himself to be the editor of the work, and he had, as it were, thrown his shield around Lambert. "Don't suppose, gentlemen," said the learned counsel, "that there was any truth in what he told you yesterday. He said he should sincerely regret the insertion of this article should it turn out to be untrue. *What, would he ruin the fame and family of an innocent and respectable gentleman, and then get off by such a whining excuse? No, he has made common cause with his reporter, and if the reporter be base, false, and infamous, the defendant is more so, for he is making a deliberate trafic in lies and slander.*" He gained his 4,000*l.* or 5,000*l.* a year by it, and law, reason, and feeling, declared that he ought to be punished for so venal, so base an attack upon the character of a man who never offended him. No other person but himself reaped profit from this slander; and the more respectable and the more popular the individual who was the object of the slander was, the more likely was it to procure an extended circulation. But the defendant had not only given publicity to the slander, but he had added to it by remarks of his own. The newspapers, too, had given publicity to the libel, and had sent it forth to the world with additional remarks. In his (Sir J. Scarlett's) opinion, founded upon a remark made by one of the witnesses, it was the height of presumption in any man to give an opinion on an operation until some explanation had been made by the operator himself, who might, perhaps, give a reason for any particular course he adopted. As to the defendant, who had given his opinion about the operation in question, he thought odds might be fairly taken that he had never performed the operation of lithotomy in his life. The learned gentleman, in allusion to the evidence of some of the defendant's witnesses, said that the only respectable witness whom the defendant had produced was Mr. Partridge. He was, from all that the learned counsel could hear, a respectable man; and if he possessed, as it was to be hoped he did, that sense of justice and of good feeling, he would profit by the lesson now offered to him,—never again to judge rashly of the skill and knowledge of any other professional man, without first giving the opportunity of explaining what difficulties might have presented themselves to him in any operation which might at first view appear to Mr. Partridge to have been unskilfully performed. With respect to the witness Gilbert, the learned counsel said, that from the hardness of his features, he could not at first believe that he was a surgeon, but rather thought that he was an ostler at some country inn. This witness had such fine sensibility, that he could not stay to see the end of the operation, but he had somehow persuaded himself that he saw the knife between the bladder and the rectum. His testimony was altogether of a curious description. Another of the defendant's excellent witnesses had given some marvellous opinions relative to surgery. One witness had never seen Mr. Cooper perform an operation well, except tying up the subclavian artery, which, he told the jury, in his opinion, might be done by accident by any ignorant person. Mr. Pearl, another witness, had actually sworn that he saw Mr. Cooper's three fingers in the wound. Now (continued the learned counsel), if this were true, there must have been an intention to murder the man, as three fingers could not have been introduced into the wound with any other possible object, and Mr. Cooper ought certainly to be tried for his life and hanged. The learned counsel was sure that the jury would indignantly repel this attempt to get up a case in order to ruin a man's reputation, and that their verdict would rather enhance his client's character with the public than lessen it. One of the defendant's own witnesses had admitted that it would be very rash in any man to find fault with an operation, unless he had communicated with the operator. There were secrets in nature which would sometimes baffle the most consummate skill. Mr. John Hunter had operated upon a man for the stone, and was an hour and a half about it. The man died, and two stones were extracted from him after his death; and notwithstanding this, was there any one who could call Mr. Hunter's skill in question? Who would dare to do so? The *Lancet*, perhaps, might dare to question it—but the

*Lancet* would dare any thing. He (Sir James Scarlett) was sure of the verdict, but he could not allow this case to go abroad even with the verdict for his client, if it could at the same time be said that that verdict was obtained merely by some defect in the defendant's evidence. He would not consent to take a verdict on such terms. For the sake of the character of his client, he should produce as witnesses in support of that character, some of the highest and most eminent men in the profession, and among others Dr. Roget, the relative of his ever-to-be lamented friend, Sir Samuel Romilly. They would all bear testimony to Mr. Cooper's skill, for, though they were competitors with Mr. Cooper, they were honourable men, and they would discard all interested motives when the honour of the profession was at stake. He should call, in addition to several surgeons of eminence from the different hospitals, one of the oldest and most respectable gentlemen in the profession—Dr. Babington, who had witnessed Mr. Cooper's practice, and who would tell the jury that, so far from Mr. Cooper being considered as an unskilful practitioner, he stood very high in the estimation of his competitors; and he would show also that the public generally held him in high repute. Dr. Babington had formed such an excellent opinion of Mr. Cooper's professional skill, that he had apprenticed his own son to him. When he had called the witnesses, and concluded his case, the defendant would have the singular advantage, and, in cases of libel, perfectly new one, of replying; but the jury, after hearing the evidence and the reply, would have only to consider what the amount of damages should be; for the verdict must be for the plaintiff. The learned counsel then made a powerful appeal to the jury for large damages. Every man was at liberty to publish what he pleased, and God forbid that that liberty should be abridged; but the liberty of the press would become one of the greatest curses unless some constitutional tribunal existed to correct its abuses. The jury were that tribunal, and they would recollect that unless a party libelled received at their hands ample reparation for the injuries inflicted on him, he might, writhing under the wounds he had received, be driven to take into his own hands that kind of "wild justice" which had been described, by Lord Bacon, in vindication of that character which his calumniator had so grossly assailed. The jury, in awarding damages in such a case as this, should mark their indignation of the defendant's conduct, and their sense of the serious injury aimed at the plaintiff's character by the libel which the defendant had thus published. He conjured them not to give an opportunity of triumph to the defendant, or to hold the plaintiff up to the scorn and contempt of the public, by giving what they might conceive to be a temperate verdict, but which malevolence and baseness would convert into a source of triumph. They would mark their sense of what was due to a character distinguished for honour, purity, and integrity, when attacked by calumny arising out of malevolent feelings and a sordid desire of gain. The learned counsel then mentioned an instance in which, in a case of less atrocious description in the City of London, 1,000*l*. damages had been awarded. Mr. Cooper's motive, however, for appealing to a jury of his country was not, he assured the jury, of a sordid nature; his object, on the contrary, was solely to wipe off the stain which the defendant had so wantonly dared to throw upon his character; and which, from the circulation of the medium employed, would be carried over a very widely extended circle. "I declare before Heaven," said the learned counsel, "that if I could but inspire you with my feelings on this occasion, no damages below those laid in the declaration should be given against this shameless libeller; and those damages, I think you are in duty, virtue, and honour, bound to give." Few medical men would have had resolution enough to come into court, on the principle that the notice thus taken of the calumny might have the effect of spreading its venom still farther. His client, however, was of too high a character to be swayed by such fears, and the greatest praise was due to him for thus courting an inquiry, and not shrinking from a task which a person of less established reputation or weaker nerve might almost excusably have been prevented from undertaking. No reparation or apology had been offered by the defendant; but, on the contrary, every pains had been taken to give to his calumnies the widest possible circulation. In a day or two they would be spread over the kingdom, and in a few weeks over the whole civilized world. The jury should ask themselves what amount of damages they would be satisfied with, if their skill, honour, and integrity, had been assailed as Mr. Cooper's had been. Would they, having a family looking to them for support, and seeing an attempt made to blast their character and future fortunes,—would they endure to be told that though they were entitled to a verdict, they must be content with moderate damages? Let them judge of Mr. Cooper's feelings by what they thought their own would be in such a case as this, and give to him such damages as they themselves would desire to have, in order to vindicate their character against so base and foul an attack. He (Sir

J. Scarlett) was sorry to have detained them so long; but he felt deeply for the individual whose character he had to defend—an individual in whose hands he would readily trust his own life; and he considered it a duty which he owed to him, as well as to the public, to make the observations which he had had the honour of submitting to their consideration.

The learned counsel, whose address occupied upwards of three hours, then put in Nos. 241 and 242 of the *Lancet*, containing the statement and "lines" alluded to in the course of his observations.

Mr. Thomas Callaway was then called and examined by Mr. F. Pollock.—He stated as follows:—I am a surgeon residing in the Borough. I am the assistant-surgeon to Guy's Hospital. I have been in the profession 17 years. I was formerly one of the pupils at Guy's Hospital, and have seen most of the operations which have taken place there. I have been present when both Sir Astley Cooper and Mr. Bransby Cooper have operated for the stone. I have operated myself six times. I have seen Mr. Bransby Cooper operate several times. I was present on the occasion in question, in the capacity of assistant-surgeon. The operation lasted, I think, about 50 minutes. I held the staff. I could not see the first incision from the position in which I was. I felt Mr. Cooper very distinctly cut into the groove of the staff which I held in my hand; I, therefore, have no doubt that the knife passed into the bladder. I was present at the examination of the body after death, and I had then no reason to think that the knife had not penetrated into the bladder. No one can form an adequate opinion of the difficulties of the operation but the operator himself. I do not think that I had the best means of ascertaining next to the operator; my situation would not enable me to judge better than a common spectator. It was evident there was considerable difficulty in feeling the situation of the stone. After the opening of the bladder the forceps were introduced. I should say that an operator would not be justified in introducing the forceps unless he was convinced by his finger that the bladder was penetrated. I have no doubt that the forceps entered the bladder after the first incision. The situation of the stone was in the anterior part of the bladder, behind the pubis, and high up. That situation accounts very satisfactorily to me for the forceps not finding the stone. From the weight of the stone, it was expected to be found in the inferior part of the bladder,—that is the place in which it is found in a large majority of cases. It was an oval flat stone. That shape readily accounts to me why it eluded the forceps. Mr. Cooper tried several forceps, straight and curved,—also a scoop and other instruments. I sounded the patient on the table before he was cut. The stone was not felt in the usual manner. It was felt on the withdrawal of the instrument, and that showed the stone to be in the anterior part. Being in the situation I have described, it would be perceptible to the sound, and yet elude the forceps. The instrument called the sound derives its name from the sound it makes on touching the stone. The patient was a stout man. During the operation Mr. Cooper asked me to try if I could reach the bladder. I think I reached the prostate gland, but am quite certain I did not reach the bladder. It was necessary to enlarge the opening. For that purpose a beak-knife was used. It required time to use it with care. A cutting gorget was afterwards used for the same purpose. I don't recollect whether a blunt gorget was used, but I think not. The gorget limits the opening, which is generally quite sufficient. If the opening had been large enough before, the gorget could do no injury, because it was introduced on the operator's finger like the knife, and if the opening was not large enough the gorget would enlarge it. In the result Mr. Cooper extracted the stone. Being in the position it was, I think he used the proper means to extract it. I think no great and unnecessary violence was used. No instruments were, I think, used, except those which were necessary to meet the apparent difficulties of the case. In my opinion, the operation was performed, under the circumstances of considerable difficulty with as much care as could possibly have been employed. The delays which occurred were, I think, entirely owing to the situation of the stone, and the difficulty in detecting it. Mr. Bransby Cooper is certainly a skilful surgeon. I have known him 20 years. I have known him from his infancy. He was with the army in the Peninsula, and also in America, as an army-surgeon. He was also at the Norwich Hospital, which is more celebrated for cases of lithotomy than any other county hospital in the kingdom. He was at Edinburgh one or two years. He was there studying medicine. I think he is skilful in his profession, and is fit to be one of the surgeons of Guy's Hospital. I attended the *post-mortem* examination. Dr. Hodgkin conducted the dissection. The bladder and rectum were examined in my presence. I saw nothing in the examination to induce me to think that the forceps had not gone directly into the bladder. If any injury had been done by violence, I must have observed it in the *post-mortem* examination. There would have been extravasation of blood. It was found that the patient was an unhealthy man. I have seen many other operations of difficulty in cases of stone, and I have known a long time employed in them. The length of time alone is not a criterion of the skill of the operator. I have seen Mr. Bransby Cooper perform the operation in about a minute. He performed one operation of this kind with success in rather more than a minute. That was since the operation in March last. I read the *Lancet*, but I have seen no report of that case in it. I was present when Mr. Cooper tied the subclavian artery; I cannot suppose it possible to perform that operation by accident. It is an operation of very great difficulty, and requires the most perfect anatomical knowledge. I think tying the subclavian artery requires more skill than the operation of lithotomy. No by-stander can understand the difficulty in extracting the stone, unless the circumstances are explained to him by the operator himself. I should not form an opinion of the operator's merit and skill, without first communicating with him on the subject of the operation.

The defendant, before proceeding to cross-examine the witness, requested that the preparation might be once more brought into court. It was accordingly handed to him, and he requested that it might be taken out of the glass, as otherwise no accurate information could be derived from it. The bladder, in a state of preservation, was accordingly taken from the glass, and handed up to the witness, whom the defendant then proceeded to cross-examine:

The *post-mortem* examination took place as usual, in public. There might have been thirty persons present. I cannot say that the stone was attached to the bladder, but there was a spot which induced me to think that that was the precise situation in which the stone was placed; the operator did say during the operation that he could not explain the cause of the difficulty; I think he had no knowledge of the situation of the stone; the loose cellular membrane is between the pubis and the bladder, and attaches the latter to the former; when the bladder is empty it is contracted, and then it might embrace the stone.

The witness proceeded. — My finger could not reach the bladder. A flat stone is more difficult to lay hold of than a round stone, and a small stone is more difficult to lay hold of than a large one. The enlargement of the opening required time to do it with care. It might take ten seconds. The cutting gorget was used only once. I did not ask the operator to explain the difficulty while the patient was under the operation. He did explain it after it was over. The patient was unbound in the course of half a minute. He was not kept bound while the "explanation" was going on. He was unbound instantly.

The defendant. — You state that Mr. Bransby Cooper is a very skilful surgeon: have you been always of that opinion?

Witness.—I have.

The defendant.—Have you not stated that it was an infamous job, placing Mr. Bransby Cooper over you at Guy's Hospital?

Witness.—No: I have no recollection of saying so.

The defendant.—Will you swear you never said so, or used words to that effect?

Witness.—I believe I could not have said so. I might, like all other disappointed candidates, have said that I ought to have been elected.

The defendant.—Have you not threatened, at different times, to publish documents to expose the corrupt system of election at Guy's Hospital?

Witness.—No; never.

The defendant.—Never to me?

Witness.—No.

The defendant.—Were you present at a dinner called the Kent Medical Dinner?

Witness.—I was at the Kent Medical Dinner about three weeks ago.

The defendant.—Did you not state to a gentleman at that dinner that Mr. Bransby Cooper was an idiot?

Witness.—No.

The defendant.—Did you not state that he was much better fitted to spend a large fortune than to be a member of our profession?

Witness.—Dr. Haslam asked me whether I had said that Mr. Bransby Cooper had a large fortune, and that he was more fit to spend it than to be a surgeon of the Hospital; and I replied that I had not said so, but that I wished he had a large fortune, and I wish so now. I said so, because if he had had a large fortune and not been a surgeon, I might have succeeded in getting my appointment. I repeat that I did not say that he was better fitted to spend a large fortune than to be a surgeon. I said I wished he was not a surgeon; but I was quite on my guard. I believe the operation lasted about 50 minutes. I don't recollect that the cutting gorget was introduced after two knives had been used.

The cross-examination proceeded further into the anatomy of the parts, but no material additional facts were elicited.

The witness was then examined further by Sir JAMES SCARLETT in support of the statement made in his address to the jury as to the mode of operating adopted by the plaintiff. The witness stated that he was of opinion that the bent forceps passed over the stone, and that it was reached only by a dextrous use of the straight forceps, aided by a considerable pressing down over the pubis. Mr. Cooper manifested no want of self possession.

In answer to some questions by a Juryman, the witness stated, that the patient was not a healthy man; he was a man of weak power, and seemed likely to sink under a great operation. He had the impression himself, and witness was of the same opinion.

Charles Aston Key, examined by Mr. SCARLETT.—I have been in the profession since 1812. I was not present at the operation in question, but was present at the examination after death. I have seen 50 or 60 operations of a similar nature. Several of them were performed by Mr. Cooper. I do not know that he lost more than an average number of patients. I thought that Mr. Cooper always performed the operation very well. I have heard the evidence of Mr. Callaway, and I concur with him in his general remarks. From what I have heard, I think I should, under similar circumstances, have adopted the very same course as that taken by Mr. Cooper. The length of time in such an operation is no impeachment of the skill of the operator. No person but the operator can be fully aware of the difficulties of the operation. I have met in my own practice several instances where great difficulty was experienced in extracting the stone after it was felt. This is caused by the contraction of the bladder. It is very muscular, and sometimes contracts and holds the stone tight. Sometimes great difficulty is experienced in consequence of the stone getting entangled in the bladder. I was not present at the operation, but I examined the body after death. There was no appearance that the operation had been performed otherwise than scientifically. If any violence had been used, I should have undoubtedly discovered it. If the forceps had gone between the bladder and the rectum, I should have perceived it—I should have found the passage through which the forceps had been passed —I should have found extravasated blood in the cellular membrane of the bladder, and have seen it in a state of slough. I saw no such appearance. The cellular membrane was perfectly sound. The other questions put to this witness were for the greater part similar to those put to the last, and produced nearly similar answers. To other questions he answered—Soon after the examination of the body, the defendant came to me in the hospital yard, where I was speaking to four or five pupils, and said, "Your straight staff will never do in a deep perineum." Knowing that the staff had nothing to do with the perineum, I said, "Sir, you know nothing at all about it; you did not perform the operation, and, besides, if you call this a deep perineum, I must say that I have operated on one twice as deep a few months back." In answer to other questions, witness said, that he had twice tied the subclavian artery. It is (where it is performed for aneurism) one of the most difficult operations in surgery. I have seen Mr. Cooper tie the subclavian artery. I never saw it better performed in my life. It is an operation that could not be performed (on the living body) by any unskilful person. It requires great anatomical knowledge, and great self-possession. I have seen Mr. Cooper perform several operations, and all of them well. I think him a very skilful surgeon.

The defendant, in cross examination, called the attention of the witness to a report in the *Lancet*, in which Mr. Cooper was highly spoken of for an operation in tying up the subclavian artery. (The plaintiff's counsel admitted this, and that the defendant had spoken in very flattering terms of the plaintiff.) Witness went on—I performed 40 operations in Guy's Hospital, but I must say, in justice to Mr. Cooper, that the greater part were young persons. I cannot say how many of Mr. Cooper's cases died. If there had been an unusual mortality, I must have noticed it, as he used an instrument which I brought into use. Witness had no recollection of any communication with the plaintiff as to the difficulties of the operation, before the publication of the report in the *Lancet*. Witness is related by marriage to Sir A. Cooper—is married to his niece.

Joseph Laundy.—I held the instruments when the operation was performed in this case. I have attended operations at St. Thomas's and Guy's Hospitals for upwards of 30 years. I have in that time seen operations performed by Sir A. Cooper, Mr. Cline, sen., and others. The longest operation for the stone I ever saw was by Mr. Cline, sen. It was an hour and 40 minutes. He extracted the stone. I have known some operations last an hour. Some of these were by Sir A. Cooper. I remember the instruments I handed to Mr. Cooper. They were not different from those used on ordinary occasions.

Cross-examined by defendant.—I cannot speak to the causes of delay in those cases which lasted more than an hour.

Dr. Hodgkin.—I am Professor of Morbid Anatomy at Guy's Hospital. I saw the body of the patient in question after death. It was that of a stout good-sized man. I examined the body, and saw no appearance of wound or bruise between the rectum and the bladder. If any such had existed, I should have seen it. James Lambert asked to see the affected parts after they were taken from the body; they were brought to him by myself or one of my assistants: *I left the room to wash my hands, and on my return Lambert showed me a hole or passage between the bladder and the rectum. I had not seen it before, and I taxed him with having made it.* I am certain, if it had existed before, I should have noticed it; if such a passage had been made in the lifetime of the subject, some coagulum would have been found in it after death. *It is my firm conviction that this passage was made after death.* I have had opportunities of seeing Mr. Cooper. I think him a fair surgeon, and decidedly a good anatomist.

Nothing of any conseqence was elicited in the cross-examination of this witness. He persevered in saying that if the hole pointed out by the defendant had existed before, he must have seen it.

Mr. Brodie, examined by Mr. CAMPBELL.—I am a surgeon, and have been a member of the Royal College since 1805. I have known Mr. Cooper in private practice. I have not seen his hospital practice. From what I have seen of him, I should say he is a skilful surgeon. From the account I have this day heard from Mr. Callaway, I should suppose the operation in question to have been a very difficult one, and that it was very ably performed by Mr. Cooper. Cases of lithotomy vary more than any other in surgery. The length of an operation is not a proof of want of skill in the operator. It would prove the difficulty of the case.

Cross-examined by the defendant.—I never saw Mr. Cooper perform an operation in my life. I attended a meeting of surgeons at Freemason's-hall in 1825, when it was considered that the publication by the *Lancet* would be injurious to those surgeons who gave public lectures. I contributed a part of the expenses for the suit in Chancery, to prevent the publication of those lectures in the *Lancet*. I do not now contribute to the support of an opposition journal to yours. I contributed to set it up, but know nothing of it since.

Re-examined.—Those who delivered public lectures felt aggrieved that their lectures should be taken from them and published, and that, too, in an incorrect form, by which discredit was brought on some of them.

Mr. Travers.—I am a surgeon. I have been in active practice for 20 years. I have heard the statement made by Mr. Callaway, and from that I see nothing which could make me suppose that the case was not skilfully performed. I have known Mr. Cooper long, and consider him a very ingenious, intelligent, and skilful surgeon. I have seen the report of the case in question in the *Lancet*. I do not believe it to be a full, fair, and correct account of what passed at the operation.

Cross-examined.—I was not present at the operation in the first instance, nor at the *post mortem* examination. I judge only by what I heard. I have performed operations for lithotomy without finding any stone, though I have no doubt that stone existed.

Re-examined by Sir JAMES SCARLETT.—I have seen Mr. Cooper perform in private practice for subclavian aneurism with great success. The report in the *Lancet* is not done in a professional manner; I should blush for the professional man who could have sent forth such a publication. I am afraid I must not designate it here by the terms which I think such a publication deserves.

Mr. Green.—I am a surgeon in St. Thomas's Hospital, and have been so for seven or eight years. I am nephew to the late Mr. Cline: I have known Mr. Cooper a long time, but have not seen him perform more than one capital operation, that was tying up the external iliac artery, which is an operation somewhat similar to that of putting a ligature on the subclavian artery. It was done with great skill, and showed a high degree of anatomical knowledge. The external iliac is that which passes over the groin, and supplies the lower limb. Mr. Green then corroborated the account given by the other surgeons as to the inferences which they drew from the evidence given by Mr. Callaway as to the skill of Mr. Bransby Cooper.

Cross-examined by defendant.—There is considerable difficulty in tying up the external iliac. In answer to other questions, witness said that he was one of those who met at the Freemasons' Tavern, on the subject of the publication of lectures in the *Lancet*. Some of witness's lectures were given, and very incorrectly. Only one of them was correctly reported. In some of the reports, he was made to state the very reverse of what he said.

Dr. Babington.—I am a physician, residing in London. I was at first educated as a surgeon at Guy's Hospital. I have known Mr. Cooper well, and have opportunities of knowing his skill as a surgeon. The best proof I can give of my opinion of his skill is, that I have placed one of my sons under him as an apprentice, and I have had no reason since to regret the choice I made.

Cross-examined by the defendant.—I do not know that it makes any difference whether a pupil be placed under one surgeon or another in the hospital. The apprentices are, I presume, all treated alike.

Dr. Roget.—I am a physician, and have given lectures on anatomy. I have had an opportunity of seeing Mr. Cooper practise, and believe him to be a man of great skill in his profession. I have read the report, and I do not think it is drawn up with the candour or skill of a professional man. I should not have believed it to have been written by a professional man.

Mr. John Morgan.—I am a surgeon of Guy's Hospital. I have seen Mr. Bransby Cooper practise, and believe him to possess great skill as a surgeon and anatomist.

Mr. Elton.—I am assistant demonstrator at Guy's Hospital. I endeavoured, at the *post mortem* examination, to reach the bladder with my finger, but could not.

Sir Astley Cooper.—I have been subpœnaed here by the defendant. I have heard the account given of the education of the plaintiff by Mr. Harrison. It is quite correct. Plaintiff was assistant to me when I was a surgeon at Guy's Hospital. He resided with me, and had constant opportunities of seeing all my public and private practice. Before this he had been two years at the Norwich and Norfolk Infirmary, where he had opportunities of being extensively acquainted with his profession. After this an appointment was obtained for him as assistant surgeon in the Artillery, and he went on the Continent. He was present at some of the most severe engagements,—the battles of Vittoria, Salamanca, and, lastly, at Toulouse; and had opportunities of becoming extensively acquainted with those surgical cases which occur in a campaign. After this he spent a year in North America, and from thence, on his return, he spent two years attending the practice in Edinburgh. Thinking, however, that the experience he had had, although it was considerable, was not sufficient to qualify him for hospital practice, I bound him apprentice to myself for six years. The plan of placing him as surgeon of the hospital was not mine; but if I had thought that he was not fit for the situation, I should most certainly have opposed his appointment. In my practice, I have often sent him as my substitute, which I should not have done if I had not thought him competent. Mr. Bransby Cooper, when he first went to the hospital, was a good anatomist and a very good surgeon, but he wanted experience in hospital practice. Still, a young man must not be crushed in the outset, or the country will be deprived of perhaps first-rate abilities. The difficulty in the present case was, that the stone was enveloped in the folds of the bladder, which did not let go its hold of it until the man became very considerably exhausted, and then it dropped, so that the straight forceps could catch it. With respect to time, it was not to be at all considered as a criterion of skill. The stone was small, and it should be known, that it was the small stones which caused the difficulty, because they were often enfolded in the bladder. No man could be a judge of the operation unless he had performed it; and no man could judge of the individual case unless he was the performer. I have been 25 years in practice, and I have in one year performed the operation of lithotomy 18 times. I should think it unkind to form an opinion of the skill displayed in a case without communicating with the operator, for I should be loth to destroy the character of another man; and unwise, because that opinion could scarcely be correct

Cross-examined by Mr. Wakley.—Sir Astley, have you never said that a good surgeon ought to be like a good general, that he should wade up to the neck in blood? Have you not said so in your lectures to your pupils?

Sir A. Cooper.—I don't know; I may have said so; I am sometimes fond of using strong expressions. I like, in short, to be understood. (Great laughter.) I think it one of the greatest evils that a man should be attacked in early life; and that he should be crushed, because he happened to have one misfortune, however capable he might be to practise in his profession. It is a power which I

think the public press ought not to have. (Laughter.)

Mr. Wakley.—Sir Astley, do you think that the interests of the public would be best consulted by having men of experience appointed as surgeons to the hospitals, and not to appoint them for the purpose of acquiring experience.

Sir Astley.—I think that every hospital ought to have an assistant-surgeon, who would thereby be prepared for the situation which he would subsequently hold.

Mr. Wakley.—Was Mr. Bransby Cooper an assistant before he became surgeon?

Sir Astley Cooper.—No, he was not; but I think it would be a good regulation to adopt in all of them.

Mr. Wakley.—What was the particular difficulty in this case?

Sir A. Cooper.—There was so little water in the bladder, that this man must have made water immediately before the operation. If the bladder was full of water, the stone would have been easily struck.

Mr. Wakley.—What is the danger to be guarded against in the operation of lithotomy?

Sir A. Cooper.—The chief danger to be guarded against is violence.

Mr. Wakley.—Supposing the stone to be felt, but enveloped in the folds of the bladder, would not the more prudent course be, after having vainly tried for ten minutes to extract it, to place the patient in his bed again?

Sir A. Cooper.—I should certainly think not, if the stone could be felt. In that case the surgeon should persevere until he got it away.

Mr. Wakley.—Have you not read Celsus, Sir Astley?—(Laughter.)

Sir A. Cooper.—I have dipped into Celsus, but I do not consider Celsus as a good surgical authority, although he is a classical one. (Much laughter.)

Mr. Wakley.—Is it not the practice in Edinburgh and Paris to put the patient to bed in such cases as I have supposed?

Sir A. Cooper.—I have studied in Edinburgh, and I have witnessed operations of lithotomy in Paris, but I never saw such a practice resorted to.

Mr. Wakley.—How long may the contraction of the bladder continue?

Sir Astley.—It might last an hour. I will give you an example—I went into the theatre at St. Thomas's Hospital, when an operation of lithotomy was being performed by a gentleman who is now in court. It was one of great difficulty. The stone was enveloped in the folds of the bladder, so that the point of it only could be felt. After nearly the lapse of an hour, he passed the instrument between the stone and the bladder; but the contraction still continued.

Mr. Wakley.—What evil could result by placing the patient in bed until the contraction was over?

Sir Astley.—Just this—what you would not like—you would have two operations instead of one to undergo. (Laughter.) If the stone was felt, the surgeon ought to persevere.

Mr. Wakley—Why is it less difficult to introduce the finger after death than before?

Sir Astley.—If you had ever put your finger into the bladder of a living subject you would know the difference. By putting your finger into the bladder of the dead subject, you would have known as much of the difficulty of this case as you do now of what is passing in the moon. (Much laughter.)

Re-examined by Sir J. Scarlett.—My nephew was a surgeon in the army, and he was my demonstrator of anatomy. He gave great satisfaction to me; and the students were so gratified with the clearness of his lectures, that they felt pleased that a man who had so easy a method of communicating knowledge should be placed in a situation where he would have the means of conveying it.

Mr. Dalrymple, the surgeon of the Norwich Hospital, deposed to the celebrity of that hospital for its operations in lithotomy, and he had himself performed the operation not less than 76 times. Had heard Mr. Callaway's description of the operation, and saw from that description no indication of want of skill on the part of the operator. Has often had similar difficulties, and has always a number of instruments resembling those that were mentioned as employed by Mr. Cooper. Believes Mr. Cooper well qualified to fill the situation of hospital surgeon. Had himself the misfortune to have a case where the stone was so large that it could not be removed, and in three hours the patient died.

Cross-examined.—In that case the stone weighed over 14 ounces.

Mr. Watson examined.—I am Secretary to the Court of Examiners of the Apothecaries' Company; the young man, Clapham, did come to be examined, in order to obtain his certificate. I have an affidavit of John Clapham, to the effect that he was 21 years of age on the 21st of April, 1828. This affidavit was necessary, in order that he should get his licence to be examined. This affidavit was sworn at a police-office in Middlesex. I don't know that Mr. Lambert did more than give a certificate of the moral character of Clapham.

Sir JAMES SCARLETT.—That is my case.

After a short pause, Mr. Wakley proceeded to reply.—He said that at the commencement of the case yesterday, the learned gentleman opposed to him expressed some apprehension lest, by the breaking down of the defendant's witnesses, the opportunity would not be afforded of bringing forward the host of evidence to the great and extraordinary skill of Mr. Bransby Cooper. The learned gentleman was then told that he might make his mind easy on that head. The result justified the declaration of the defendant. His witnesses had not broken down, and no charges against Mr. Cooper had been answered. A verdict must therefore be given for the defendant. After referring to the origin and progress of the *Lancet*, the defendant declared that the proceedings of that day were the most peculiar that ever took place. One would have supposed, from the parade with which this case was heralded, that the report in the *Lancet* was a fabrication—that no such operation was ever performed—and that it had no foundation from beginning to end, except in the malevolent mind of the defendant. One of the witnesses for the plaintiff admited that there were 200 spectators, and yet, with all the influence of Mr. Bransby Cooper, he was only able to produce one single spectator to rebut the charge made against him. The plaintiff sought every where but in the proper place for witnesses; he had surgeons from St. Thomas's Hospital, he had Sir Astley Cooper from Conduit Street, and it was to be wondered that the Emperor of Morocco was not also thrust into the box. With reference to the influence which Sir Astley Cooper had in the appointment of surgeons to Guy's Hospital, he put it to the Jury whether Sir Astley was entitled to recommend any man to the care of patients as he had done his own nephew in this instance. This hospital belonged not to the directors, but to the poor, for whose use public funds had been granted. Mr. Callaway was elected an assistant surgeon, and Sir Astley Cooper consulting surgeon, on the same day that Mr. Bransby Cooper was appointed to the situation of surgeon. It was a saying of John Hunter, that bad surgeons made work for good ones; and that good surgeons would starve but for bad ones. Perhaps it was in reference to the operations of Mr. Bransby Cooper that these two supernumeraries have been appointed.

The defendant here became so exhausted, that he retired from the court for a few minutes. On his return, he observed again upon the absence of witnesses in reply to the charge, who had been present at the operation. Leaving out of consideration the technicalities of pleading, the defendant thought himself fully entitled to a verdict if the report were substantially correct. Could it be pretended that Mr. Cooper was a skilful surgeon when he was unable to explain the causes of the difficulty that presented itself? Could he (Mr. Cooper) be said to have had presence of mind when, whilst he was plunging his instruments into the unfortunate patient, he openly declared that he could not find the reason for not being able to extract the stone? The poor man repeatedly called on them and implored them to loose him—he knew the torture of his complaint, he felt the torture which he was then undergoing, still he cried to be let go. "No," said Mr. Bransby Cooper, "my reputation is concerned, I must take out the stone, even though you should die." He did die. The defendant most solemnly protested to God that he had no ill will towards Mr. Cooper; he believed him to be a most amiable private character; but as a surgeon—as a surgeon of a public hospital—as a public functionary—he denounced him to be ignorant and incompetent. Let the jury weigh well the evidence, and if they felt that they, if afflicted in a similar way, would not suffer Mr. Bransby Cooper to operate on them, then they must find their verdict for the defendant.

At the conclusion of the defendant's address, which was delivered with great animation and effect, he was applauded by a number of persons in the remote part of the court.

This ebullition of feeling was checked by a very decided intimation from Lord Tenterden that he would commit the parties to custody, if they persevered in it.

The LORD CHIEF JUSTICE, in summing up the evidence, observed, that if the jury believed that this report was a *bonâ fide* account of the transaction, they must, without reference to its effect upon the character of Mr. Cooper, find a verdict for the defendant. If, however, it was erroneous or falsely coloured, intending to injure the professional reputation of the plaintiff, they must find a verdict for him. His Lordship, in the course of his address, pointed the attention of the Jury to what seemed to be the general impression of the witnesses for the plaintiff—that the report was drawn up in an unprofessional manner—that it was illiberal and uncandid in its remarks—and that, in point of fact, no report of a case tending to prejudice the operator ought to have been sent before the public without the party making it having consulted the operator himself, as he alone was the best judge of the difficulties of the particular case in which he was engaged. The question, His Lordship observed, was one of great importance, not only to the individual, but to the public, and called for the serious consideration of the jury. The real question they had to decide was, whether or not the operation was performed in a skilful manner. One of the grounds on which it was stated not to be so was the length of time taken in it; but on this subject they had heard from some of the most skilful surgeons of the day that shortness or length of time was by no means a test of the skill of the operator, but depended upon the difficulties which might or might not present themselves in each particular case. If on the whole they believed that Mr. Cooper had shewn professional skill in the performance of that operation, and was generally fitted for the duties of the station he now filled, they would find a verdict for him, with damages sufficient—not, as had been said by the counsel of the plaintiff, to mark their indignation at the charge, but, to meet the justice of the case, after giving it the most mature and temperate consideration.

At a quarter to nine the Jury retired to consider their verdict, and at near eleven o'clock they returned to court, finding a verdict for the plaintiff—Damages 100*l*.

There was a slight burst of applause in the court on hearing the announcement, which, however, was speedily suppressed by the officers.

This trial seemed to excite very general interest, and the court was crowded in every part up to the very last moment.

